# Stress-associated protein OsSAP5 regulates rice heading date through interacting with OsGF14c in rice

**DOI:** 10.3389/fpls.2025.1589989

**Published:** 2025-09-09

**Authors:** Xiaobo Zhu, Min Chen, Zhang Dong, Yihan Liu, Qingshan Mu, Farwa Basit, Jiaxin Liu, Jin Hu, Yajing Guan

**Affiliations:** ^1^ Hainan Institute, Zhejiang University, Sanya, China; ^2^ Institute of Crop Science, Zhejiang Key Laboratory of Crop Germplasm Innovation and Utilization, College of Agriculture and Biotechnology, Zhejiang University, Hangzhou, China; ^3^ Department of Biology, College of Science and Technology, Wenzhou-Kean University, Wenzhou, China

**Keywords:** rice (*Oryza sativa*), OsSAP5, heading date, OsGF14C, E3 ubiquitination ligase

## Abstract

Heading date, a critical agronomic trait determining rice regional adaptation and yield potential, is regulated by complex genetic networks. Although stress-associated proteins (SAPs) are well-documented mediators of abiotic stress responses, their roles in reproductive development remain poorly characterized. Here we demonstrate that stress-associated protein 5 (OsSAP5) functions as a positive regulator of heading date in rice. Loss-of-function *ossap5* mutants exhibited significant heading delay under natural short day condition, accompanied by marked downregulation of florigen genes *Hd3a* and *RFT1* and subsequent suppression of MADS-box genes in the shoot apical meristem (SAM). A similar decrease in expression of florigen genes was observed in *OsGF14c* overexpression lines under short day condition. The molecular and biochemical assays confirmed that OsSAP5 interacts with OsGF14c. Notably, OsSAP5 had E3 ubiquitin ligase activity and might promote the ubiquitination of OsGF14c. Therefore, OsSAP5 regulates heading date by interacting with OsGF14c under short day condition.

## Introduction

Rice (*Oryza sativa*) is one of the most important crops in the world, and heading date is one of the main factors affecting the yield (Cai et al., 2021). Rice is a typical short day (SD) plant, the heading date of which is promoted under SD condition and delayed under long day (LD) condition. *Hd3a* and *RFT1*, FT-like genes, as the florigen play critical roles in regulating heading date in rice (Komiya et al., 2008; Kojima et al., 2002). Hd3a acts as mobile florigens moving from leaves to the shoot apical meristem (SAM), where it forms the florigen activation complex (FAC) with 14-3–3 and OsFD1 under SD condition. RFT1, as the same florigen in LD condition, performs a similar function (Tamaki et al., 2007; Komiya et al., 2009; Taoka et al., 2011; Peng et al., 2021). FTL12, a member of FT-like family, interacts with OsGF14b and OsFD1 to form the florigen repression complex (FRC) by competing with Hd3a for binding OsGF14b (Zheng et al., 2023). The RICE CENTRORADIALIS (RCN) is a rice TFL 1-like protein that also competitively binds to 14-3–3 with Hd3a and form a FRC (Wang et al., 2020). OsbZIP42/HBF1 and OsbZIP9/HBF2, two paralogs of OsFD1, form a complex with Hd3a in leaf cells, which represses *Hd3a* expression under SD and LD conditions. HBFs could also interact with RFT1 via OsGF14c as the bridge to form a complex in leaf cells, which also represses *RFT1* expression under SD and LD conditions (Brambilla et al., 2017).

Under SD condition, the pathway of *OsGI*-*Hd1*-*Hd3a* promotes rice heading, which is conserved with the *GI*-*CO*-*FT* pathway in *Arabidopsis* (Zhu et al., 2023). Hd1 promotes heading date by inducing directly *Hd3a* expression (Kojima et al., 2002). OsGI induces *Hd1* expression under SD and LD conditions (Hayama et al., 2003). Under LD condition, the pathway *Ghd7*-*Ehd1*-*Hd3a*/*RFT1* is the main pathway to promote rice heading (Zhu et al., 2023). Ehd1 is a special gene in rice and induces *Hd3a* and *RFT1* directly in a special rice variety that lacks the Hd1 function under SD condition (Doi et al., 2004). The expression of *Ghd7* increases as the day progresses, and Ghd7 represses the expression of *Ehd1* (Xue et al., 2008; Itoh et al., 2010). Hd1 also suppresses *Hd3a* expression under LD condition (Hayama et al., 2003).

The A20/AN1-type zinc finger protein, also called stress-associated protein (SAP), is well known to play significant roles in stress response and plant development. In mammals, the A20 family proteins regulate NF-kB, a tumor necrosis factor, with activation through the ubiquitin–proteasome system (Heyninck et al., 1999). In plants, there are 18 SAP protein family members in rice and 14 members in *Arabidopsis*, namely, OsSAP1–18 and AtSAP1-14 (Vij and Tyagi, 2006). *OsSAP1* is first reported and overexpression lines in tobacco conferred tolerance to cold, dehydration, and salt stress at seed germination/seedling stage (Mukhopadhyay et al., 2004). AtSAP5 shows a strong homology to OsSAP1 and has E3 ubiquitin ligase activity. Plant that overexpress *AtSAP5* has increased tolerance to salt stress, osmotic stress, and water deficit (Kang et al., 2011). AtSAP5 promotes AtMBP-1, a *c*-myc binding protein, degradation by ubiquitin-dependent proteasome pathway (Choi et al., 2012; Feo et al., 2000; Kang et al., 2011). TaSAP5 overexpression in *Arabidopsis* and wheat seedlings increased their drought tolerance (Zhang et al., 2017). DREB2A INTERACTING PROTEIN1 (DRIP1) and DRIP2, as E3 ubiquitin ligase, interact with drought response transcription factor DEHYDRATION-RESPONSIVE ELEMENT BINDING PROTEIN2A (DREB2A) (Qin et al., 2008). TaSAP5 interacts with TaDRIPs, leading to their subsequent degradation through the 26S proteasome pathway and then increasing the level of DREB2A protein and its downstream genes (Zhang et al., 2017). Plants that overexpress *AtSAP9* delayed the flowering time by about 4 days under LD condition, and the expression analysis showed that the expression of *CO*, *SOC*, and *FT* significantly decreases (Kang et al., 2017). The effector of *AtSAP8*-overexpressing in tobacco showed early flowering time by about 9 days (Lin et al., 2023).

Ubiquitination is a posttranslational modification process. Ubiquitin/26S proteasome system (UPS) plays important roles in plants to cope with environmental changes or in plant development (Linden and Callis, 2020). This system needs ubiquitin-activating enzyme (E1), ubiquitin-conjugating enzyme (E2), and ubiquitin ligase (E3). Ubiquitin binds to E1, and E1 transfers ubiquitin to E2, which binds with E3. Then, E3 facilitates the transfer of ubiquitin from E2 to the substrate. The consequences of polyubiquitinated substrate become a target for the 26S proteasome for subsequent degradation (Al-Saharin et al., 2022). The abundant E3 ubiquitin ligase determines the plant development and response to environment changes, as E3 can bind to the substrate directly, and different substrates have different functions in the plant (Mazzucotelli et al., 2006).

In our laboratory, the previous study proved that *OsSAP5*-overexpressed lines in *Arabidopsis* increase heat tolerance during seed germination (Chen et al., 2021a). In this study, we provided evidence that OsSAP5 is an E3 ubiquitin ligase and is involved in positively regulating heading date under natural short day condition. OsSAP5 might also promote OsGF14c degradation by UPS. Consistent with this, the florigen *Hd3a* and *RFT1* expression was lower in *OsSAP5* mutants and in the *OsGF14c* overexpression lines compared to NIP.

## Materials and methods

### Plant materials and growth conditions

NIP (*Oryza sativa* L*. Japonica* cv. *Nipponbare*) was used to generate transgenic lines. NIP, *ossap5* mutants, and *OsSAP5* overexpression lines were grown at the Sanya transgenic experimental base of Zhejiang University, China. The natural long day (NLD) was in Changxing, Zhejiang, China (119.91° E, 31.02° N). The natural short day (NSD) was in the Sanya, Hainan, China (109.5° E, 18.5° N). The germinated seed was grown for 15 days under control short day (CSD) (10-h light and 14-h dark at 28 °C) and control long day (CLD) (14-h light and 10-h dark at 28 °C), respectively.

### Vector construction and rice transformation

To construct the CRISPR/Cas9 vector for *OsSAP5* or *OsGF14c*, the predictions made by CRISPR-GE (http://cbi.hzau.edu.cn/cgi-bin/CRISPR2/CRISPR) were used to select two guide sequences with a low off-target rate (Xie et al., 2017). The gRNA framework with the guide sequence was inserted into the pYLCRISPR/Cas9Pubi-H empty vector to produce the CRISPR/Cas9 recombinant vector. *OsSAP5* or *OsGF14c* CDS sequences were amplified by PCR using cDNA as the template and then inserted into the pRHVcMYC for overexpression vector construction. The 2.0-kb *OsSAP5* promoter sequences (i.e., sequences upstream of the ATG start codon) were amplified by PCR using genomic DNA as template and then inserted into pDX2181 vector to control the expression of the GUS-encoding gene. The promoter sequences replaced the 35s promoter sequences of pC1300-35s-EGFP vector by using restriction enzymes *Nco*I and *Sac*I, and then the CDS sequences were inserted into the pC1300-*pro*OsSAP5-EGFP vector. All primers used to construct the recombinant vectors are listed in **Supplementary Table S3**.

### Histochemical analysis of GUS activity

For the GUS staining analysis, leaf blades, leaf sheath, SAM, stems, and young panicles were collected from transgenic plants of *pOsSAP5::GUS* at 30-day-old seedling and the heading stage. The GUS activity was detected using the GUSblue kit (Huayueyang, Beijing, China) according to the manufacturer’s manual. The samples were destained with pure ethanol and then examined using a dissecting microscope (Leica PLANPOFOV3.6). All primers used to construct recombinant vectors are listed in **Supplementary Table S3**.

### Fluorescence microscope observation of the transgenic OsSAP5-EGFP

The leaf sheath of 15-day-old seedlings was cut 2 to 3 cm by using a scalpel, thus obtaining interesting tissue (the seedling leaf sheath of 2~3 cm). The tissue was put into the embedding cassette with the fixative (Tissue-Tek O. C. T. Compound) for frozen 30 min at -20°C, making sure that the tissue was fully surrounded by the fixative. Then, a machine (CryoStar NX50 OPD) was used to cut the section with 50-μm thickness. Finally, the section of the interact tissue was observed using a fluorescence microscope (LEICA DFC7000 T). All primers used to construct the recombinant vectors are listed in **Supplementary Table S3**.

### Subcellular localization

*OsSAP5* or *OsGF14c* coding sequence was inserted into pC1300-35s-EGFP for subcellular observation. AtPIPT2A-mCherry was used as cell membrane marker (Nelson et al., 2007). The nucleus marker was histone H2B-mCherry. The OsGF14c sequence was replaced with the H2B sequence of the H2B-mCherry nuclear marker vector by using restriction enzymes *Kpn*I *and Bam*HI. *Agrobacterium tumefaciens* GV3101 cells were transformed with the pC1300-35s-EGFP empty vector or the pC1300-35s-OsSAP5-EGFP recombinant vector for the subsequent infiltration of healthy *N. benthamiana* leaves. Fluorescent signals were detected using the Leica/STELLARIS 5 laser scanning confocal microscope. For green fluorescence observation, the excitation wavelength was 488 nm and the emission wavelengths were 520 to 540 nm; for mCherry fluorescence observation, the excitation wavelength was 561 nm and the emission wavelengths were 610 to 630 nm. The primers used for the subcellular vectors are listed in **Supplementary Table S3**.

### Rhythmic expression analysis

The germinated seed was transferred to the soil matrix for growing 15 days in a growth chamber with SD condition (10 h light and 14 h dark at 28°C) or with LD condition (14 h light and 10 h dark at 28°C). The leaves of each line were harvested every 4 h for 20 h. For each time point, the leaves from three different individuals were collected as biological replicates.

### RNA extraction and quantitative real-time PCR

Total RNA was isolated using Trizol solution (Vazyme, Najing, China). cDNA was synthesized from 1 μg of total RNA using the cDNA synthesis kit with a gDNA wiper (Vazyme, Najing, China). One microliter of cDNA was used for real-time-PCR analysis with gene-specific primers using SYBR Green PCR master mix in a LightCycler480 system (Roche). The 2^−ΔΔCt^ method described was used for the analysis of relative gene expression (Livak and Schmittgen, 2001). Three biology replicates were performed. All of the qRT-PCR primers are listed in **Supplementary Table S3**.

### Bimolecular fluorescence complementation assay

The *OsSAP5* coding sequence was PCR-amplified and inserted into pSPYCE-35s vector, and the *OsGF14c* coding sequence was PCR-amplified and inserted into pSPYNE-35s vector (Wang et al., 2023). *Agrobacterium tumefaciens* GV3101 cells were transformed with the recombinant vector, which were combined (1:1, v/v) for the subsequent infiltration of healthy *N. benthamiana* leaves. Fluorescent signals were detected using Leica/STELLARIS 5 laser scanning confocal microscope. The laser used was similar to the methods of subcellular localization. The primers used for bimolecular fluorescence complementation (BiFC) vectors are listed in **Supplementary Table S3**.

### Luciferase complementation imaging assay

The plasmids of pCAMBIA1300-cLUC and pCAMBIA1300-nLUC were described in the paper (Chen et al., 2008). The *OsSAP5* coding sequence was PCR-amplified and inserted into pCAMBIA1300-cLUC vector, and the *OsGF14c* coding sequence was PCR-amplified and inserted into pCAMBIA1300-nLUC. *Agrobacterium tumefaciens* GV3101 cells were transformed with the recombinant vector, which were combined (1:1, v/v) for the subsequent infiltration of healthy *N. benthamiana* leaves, and incubated in the growth room (25°C light/23°C dark) for 48 h before the LUC activity measurement. For the CCD imaging and LUC activity measurement, 1 mM luciferin was sprayed onto the leaves. The cooled CCD imaging apparatus was used to capture the LUC image. Each data point contains at least four replicates, and three independent experiments were carried out. The primers used for LCI vectors are listed in **Supplementary Table S3**.

### Pull-down assay

*OsSAP5* and *OsGF14c* coding sequences were inserted into pET32a and pGEX-4T-1 vectors, respectively. *Escherichia coli* BL21 (DE3) cells were transformed with the recombinant vectors for the expression of GST-OsGF14c or His-OsSAP5 proteins. A pull-down assay was performed for 2 h at 4°C using GSTSep Glutathione Agarose Resin (GST) particles (YEASEN, Shanghai, China) in binding buffer (25 mM Tris-HCl [pH = 8.0]; 150 mM NaCl; 1/2,000 (v/v) NP-40; 1/20 (v/v) glycerinum). Finally, the samples were boiling at 100°C for 10 min and immunoblotted using anti-GST (Abmart, 1:1,000) and anti-His (Abmart, 1:1,000) mouse monoclonal antibody. The primers used for the pull-down vectors are listed in **Supplementary Table S3**.

### Co-immunoprecipitation assay

*OsSAP5* and *OsGF14c* coding sequences were inserted into pRTVcHA and pRTVcGFP vectors to generate p35s::OsSAP5-HA and p35s::OsGF14c-GFP, respectively. To transiently express these recombinant vectors, the vector combinations were co-transformed into 100 mL of rice protoplasts using a PEG-mediated transfection method as described previously (Zhang et al., 2011). After incubation at 25°C for 14 h, the transfected protoplasts were pelleted at 200 g for 3 min. The pellets were resuspended in 500vmL protein extraction buffer (50 mM Tris–HCl [pH 8], 500 mM sucrose, 1 mM MgCl_2_, 10 mM EDTA, 5 mM DTT, 1× PMSF, and 1× protease inhibitor cocktail) and then incubated at 4°C for 1 h with vortexing. After centrifugation at 13,000 *g* for 10 min at 4°C, 20 μL anti-GFP affinity beads 4FF (Smart-Lifesciences, Changzhou, China) was added to the supernatant with rotation at 4°C for 2 h. The beads were then washed with protein extraction buffer three times and boiled with 100 μL 1× SDS-PAGE loading buffer (Beyotime, Shanghai, China) for 10 min at 100°C to elute bound proteins from the beads. The proteins were then separated in 10% SDS-PAGE gels and detected by western blot analysis using anti-GFP antibodies (Abmart 1:1,000) and anti-HA antibodies (Abmart, 1:1,000). The primers used for the Co-IP vectors are listed in **Supplementary Table S3**.

### *In vitro* ubiquitination assay

Ubiquitination assay was performed as previously described with some modifications (Wang et al., 2017). Briefly, 1 μg His-OsSAP5 was incubated in a 20 μL reaction mixture containing 1 M Tris-HCl (pH 7.5), 200 mM MgCl_2_, 40 mM DTT, 100 mM ATP, 50 ng E1 (R&D systems, E-305-025), 150 ng E2 (Sigma-Aldrich, UbcH5b), and 1 μg ubiquitin (R&D systems, HA-Ub) and incubated at 30 °C for 1.5 h. The reaction was stopped by adding 1× SDS-PAGE loading buffer (Beyotime, Shanghai, China) and boiling at 100°C for 10 min. The proteins were then separated in 10% SDS–PAGE gels and detected by western blot analysis using anti-HA antibodies (Abmart, 1:1,000).

### *In vitro* degradation assay

A cell-free protein degradation assay was performed as previously described (Spoel et al., 2009). The total protein of NIP 30-day-old seedling was extracted from cultured cells with proteolysis buffer (25 mM Tris-HCl, pH 7.5, 10 mM MgCl_2_, 10 mM NaCl, 10 mM ATP, and 5 mM DTT). Then, total protein and His-OsGF14c protein were incubated with the inhibitors PMSF (4 μM), MG132 (150 μM, Yuanye Biotechnology Co. Ltd., Shanghai, China), or organic solvent DMSO at 25°C, with the sample taken every 30 min for 2 h. Finally, the samples were boiling at 100 °C for 10 min after adding 1× SDS loading buffer; then, the proteins were separated in 10% SDS–PAGE gels and detected by western blot analysis using anti-His antibodies (Abmart, 1:1,000).

### IP-MS analysis

The seedlings of NIP and OsSAP5-MYC were collected and ground into fine powder in liquid nitrogen. The total protein was isolated with Co-IP buffer, and then the homogenate was centrifuged at 13,000 rpm for 10 min at 4°C. The supernatant was collected and incubated with Anti-c-Myc Affinity Beads (Smart-Lifesciences, Changzhou, China) for 4 h at 4°C. After washing, the immunoprecipitated protein mixture was eluted and analyzed by LC-MS/MS using a Q Exactive HF-X System (Thermo Fisher). The spectrum data were searched against the RGAP database using Protein Prospector (Chen et al., 2021b).

### Dual LUC reporter assays

The *OsSAP5* coding sequence was inserted into pRT-BD-GAL4 plasmid. The empty BD-GAL4 served as negative control, and BD-GAL4-VP16 was the positive control. The reporter plasmid was the pGreenII-0800-5×UAS_35S_LUC vector. The effector and reporter plasmids were co-transformed into rice protoplasts. After 12 h of incubation in the dark, the protoplasts were collected and immediately utilized for LUC assays. Untreated protoplast populations were used as controls. The relative LUC activity was quantified using the Dual Luciferase Assay Kit (Promega) and then calculated as the ratio of fLUC to rLUC (fLUC/rLUC). Renilla LUC was used as an internal control. Three independent transformations were performed for each sample. The primers used for subcellular vectors are listed in **Supplementary Table S3**.

### Expression of *OsMADS* genes in rice protoplasts

Promoter sequences of about 2,264 bp of *OsGF14c* replaced the Ubiq promoter sequences in pRTVcGFP vector by using restriction enzymes *Pst*l and *Eco*RV. Then, the *OsFD1* and *OsGF14c* coding sequences were inserted into the vector with pOsGF14c sequences to generate *pOsGF14c::OsFD1* and *pOsGF14c::OsGF14c*. The promoter sequences, about 2,060 bp of *Hd3a*, replaced the Ubiq promoter sequences in pRTVcGFP vector by using restriction enzymes *Pst*l and *Eco*RV. Then, the *OsGF14c* coding sequence was inserted into the vector with pHd3a sequences to generate *pHd3a::OsGF14c*. The Hd3a coding sequence was inserted into pRTVcMyc to generate *p35s::Hd3a*. The different combination of effector was transformed into rice protoplasts by the methods of CO-IP. After 60 h of incubation, the checking of *OsMADS* expression was similar to the methods in “RNA extraction and quantitative real-time PCR”. The primers used for subcellular vectors are listed in **Supplementary Table S3**.

## Results

### OsSAP5 positively regulates heading date in rice

**Figure 1:**
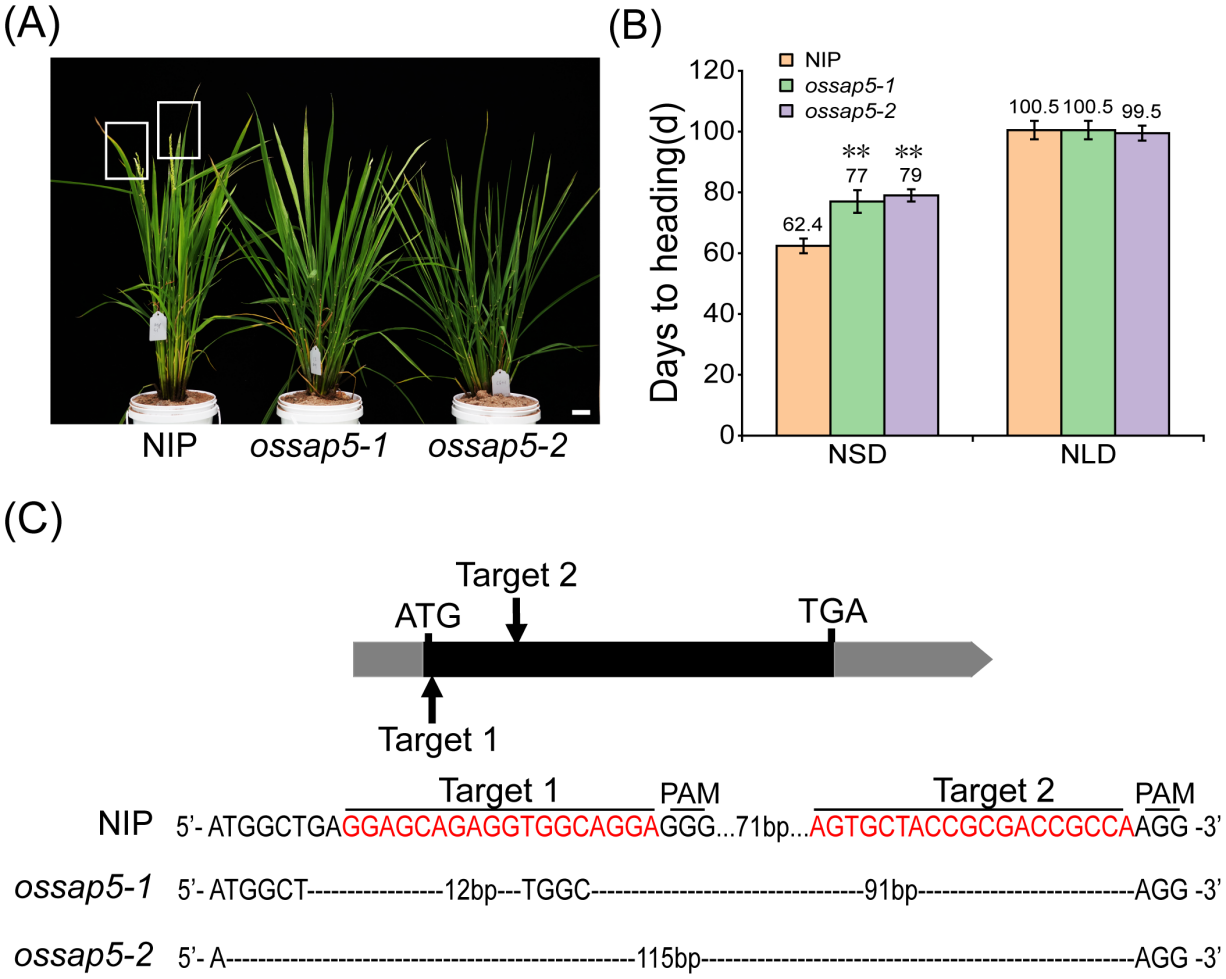
Heading date of *OsSAP5* mutants. **(A)** Heading phenotype of NIP (*Oryza sativa* L.) and *OsSAP5* mutants under NSD condition. Bar = 10 cm; *ossap5–1* and *ossap5–2* represent two *OsSAP5* mutants. The rectangular box indicates rice heading. **(B)** Days to heading of NIP, *OsSAP5* mutants under NLD and NSD conditions. Values are presented as means ± SD (*n* = 15, **P < 0.01; Student’s *t*-test). NSD means natural short day. NLD means natural long day. **(C)** Generation and identification of two *OsSAP5* mutants generated by CRISPR/Cas9 genome-editing approach. The target 1 and target 2 sites and the protospacer adjacent motif (PAM) are indicated. The black dash indicates the deletion of a base pair.

**Figure 2:**
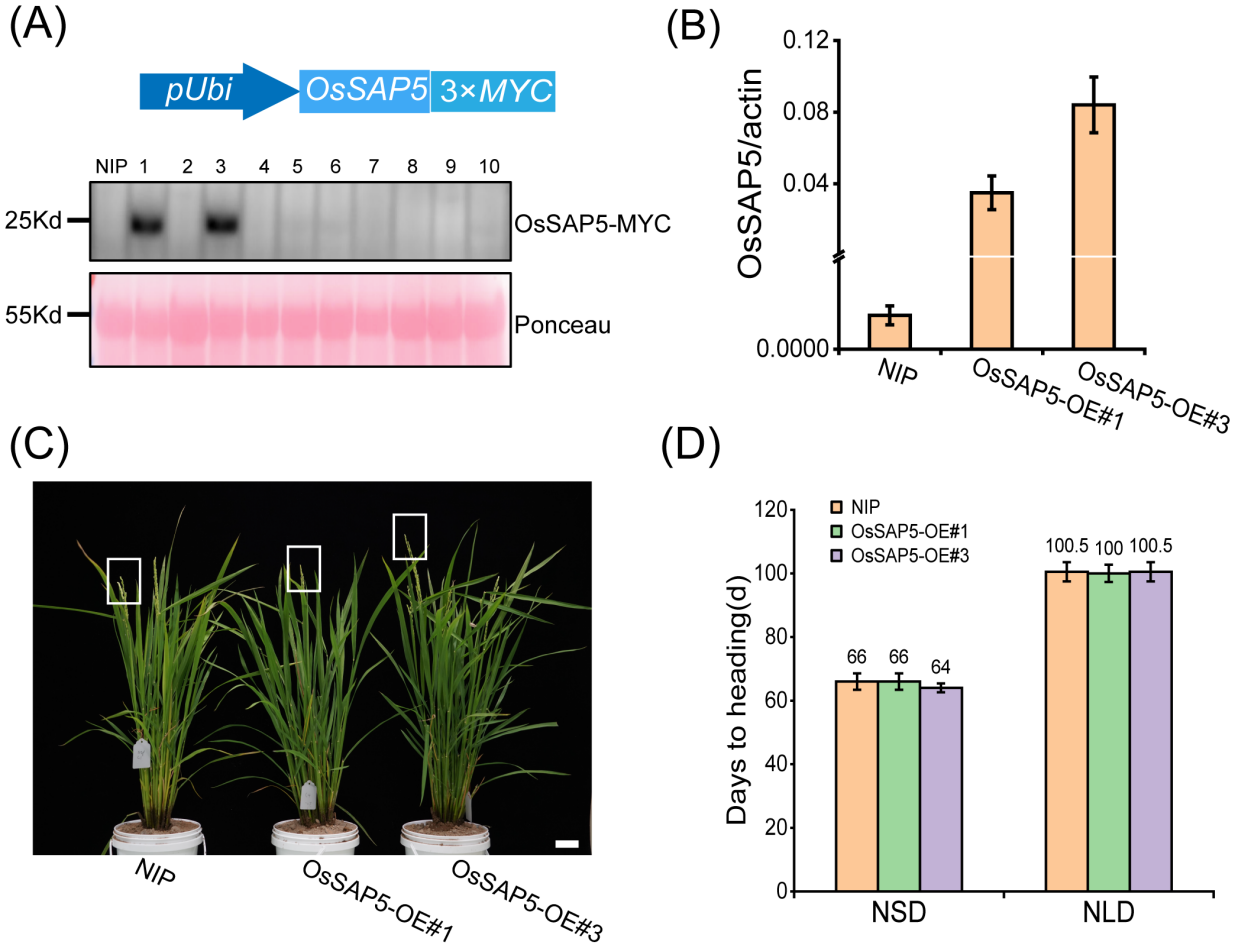
Heading date of *OsSAP5* overexpression lines. **(A)** MYC-tag immunoprecipitate of *OsSAP5* overexpression plants. A total of 10 protein samples are detected with anti-MYC antibodies with loading as shown by Ponceau S staining for RUBISCO. **(B)** qRT-PCR analysis of the expression levels of *OsSAP5* in NIP and OsSAP5 overexpression lines. Values are presented as means ± SD (*n* = 3). OsSAP5-OE#1 and OsSAP5-OE#3 indicate two *OsSAP5* overexpression lines, respectively. **(C)** Heading phenotype of NIP and *OsSAP5* overexpression lines under NSD condition. Bar = 10 cm. The rectangular box indicates rice heading. **(D)** Days to heading of NIP, *OsSAP5* overexpression lines under NLD and NSD conditions. Values are presented as means ± SD (*n* = 15).

In our previous study, *OsSAP5*-overexpressed lines in *Arabidopsis* enhanced the tolerance to drought during seed germination (Chen et al., 2021a), but the function of *OsSAP5* remains unknown in rice. The *ossap5*-knockout mutants were generated in the *japonica* rice cultivar Nipponbare background using the CRISPR/Cas9 genome editing approach. Two independent homozygous lines (*ossap5-1* and *ossap5-2*) were selected for further study. Both mutant lines showed the deletion of long fragment bases in *OsSAP5* coding sequence (**Figure 1C**). Therefore, OsSAP5 protein function was disrupted. The seedlings of two mutant lines showed lower tolerance to heat stress and drought compared to NIP (data not shown). In addition, in the natural short day (NSD) field, *ossap5-1* and *ossap5-2* delayed about 15 days in heading date compared to NIP, but no significant differences were observed under natural long day (NLD) condition (**Figures 1A, B**; **Table 1**). The overexpression construct (*pUbi::OsSAP5*-*MYC*) was transformed into Nipponbare, and two homozygous lines (OsSAP5-OE#1 and OsSAP5-OE#3) with MYC tag, which had increased *OsSAP5* expression, were selected for further study (**Figures 2A, B**; **Supplementary Figure S1**). Both overexpression lines showed the same heading date compared to NIP under both NSD and NLD conditions (**Figures 2C, D**; **Table 2**).

**Table 1:** Days to heading of NIP, *ossap5-1*, *ossap5-2* under NSD and NLD conditions.

Genotype	NIP	*ossap5-1*	*ossap5-2*	Time (year)
Days to heading				
NSD				
	83.6 ± 1.50	95.0 ± 1.80**	96.5 ± 0.71*	2023
	62.4 ± 2.40	77.0 ± 3.70**	79.0 ± 2.6*	2024
NLD				
	100.5 ± 3.00	100.5 ± 3.00	99.5 ± 2.45	2023
	91.0 ± 2.16	91.5 ± 1.58	92.5 ± 1.87	2024

Values are presented as means ± SD (*n* = 15, *P<0.05, **P<0.01; Student’s *t*-test).

**Table 2:** Days to heading of NIP, OsSAP5-OE#1, and OsSAP5-OE#3 under NSD and NLD conditions.

Genotype	NIP	OsSAP5-OE#1	OsSAP5-OE#3	Time (year)
Days to heading				
NSD				
	83.6 ± 1.50	83.0 ± 1.80	83.3 ± 2.07	2023
	66.0 ± 2.50	66.0 ± 2.60	64.0 ± 1.40	2024
NLD				
	100.5 ± 3.00	100 ± 2.70	100.5 ± 3.00	2023
	91.0 ± 2.16	92.0 ± 1.87	92.5 ± 1.87	2024

*n* = 15.

*ossap5*-*CRISPR* exhibited a higher seed setting compared to NIP, while maintaining comparable plant height, tiller number, and yield per plant under NSD condition (**Supplementary Table S1**). However, *OsSAP5*-overexpressing lines showed significantly reduced seed setting and decreased yield per plant relative to NIP (**Supplementary Table S1**). These findings demonstrate that *OsSAP5* positively regulates heading date and negatively regulates seed setting under NSD condition.

### *OsSAP5* influences florigen *Hd3a* and *RFT1* expression

**Figure 3:**
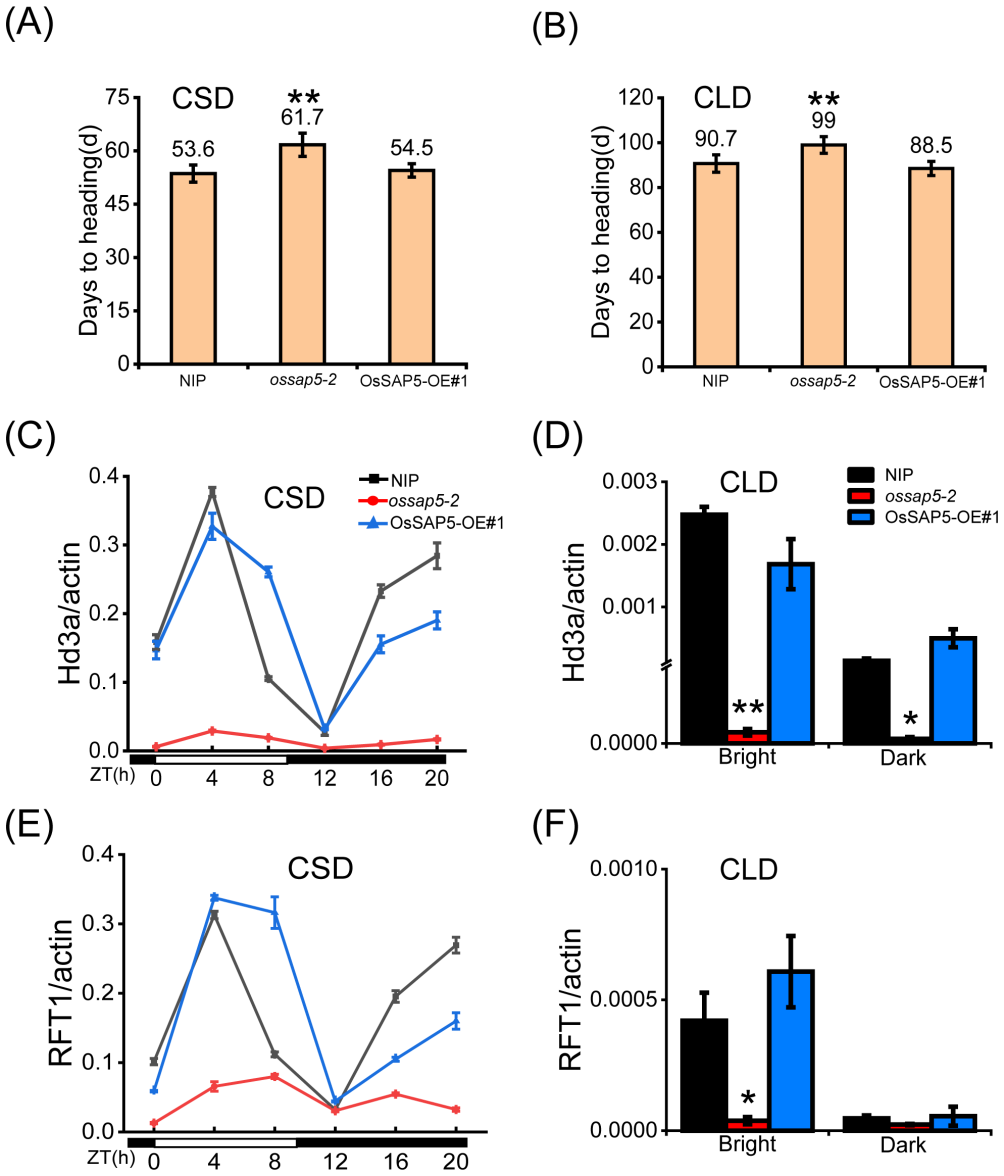
Heading date of *OsSAP5* transgenic material under CSD and CLD conditions. **(A, B)** Days to heading of NIP, *ossap5–2* and OsSAP5-OE#1 under CSD and CLD conditions. Values are presented as means ± SD ((*n* = 15, **P < 0.01; Student’s *t*-test). CSD means control short day. CLD means control long day. **(C, E)** Rhythmic expression of *Hd3a* **(C)** and *RFT1* **(E)** in NIP, *ossap5-1*, and OsSAP5-OE#1 under CSD conditions. ZT is the means of Zeitgeber time. **(D, F)** Expression of *Hd3a* **(D)** and *RFT1* **(F)** in NIP, *ossap5-1*, and OsSAP5-OE#1 under CLD conditions. Values are presented as means ± SD ((*n* = 3, *P < 0.05; **P < 0.01; Student’s *t*-test).

Under the chamber conditions, the *ossap5*-*CRISPR* delayed heading date about 7 days and 9 days compared to NIP under control SD (CSD) and control LD (CLD) conditions, respectively (**Figures 3A, B**). However, the *OsSAP5*-overexpression lines showed no significant difference in heading date (**Figures 3A, B**). To investigate the effect of *OsSAP5* on the flowering-related genes, the expression of two key pathway genes (*GI*, *Ghd7*, *Ehd1*, *Hd1*, *Hd3a*, *RFT1*) related to heading date and FAC member genes (*OsGF14b*, *OsGF14c*, *OsFD1*) was examined in NIP, *ossap5–2* and OsSAP5-OE#1. The expression of these genes did not change significantly, but florigen genes *Hd3a* and *RFT1* were obviously decreased in *ossap5–2* under CSD condition, with no difference in OsSAP5-OE#1 (**Figures 3C, E**; **Supplementary Figure S2**). The expression of *Hd3a* and *RFT1* was too low to be detected at each ZT (Zeitgeber time) points under CLD condition, so we examined changes in the expression of *Hd3a* and *RFT1* at two points (bright and dark). The results showed the expression of *Hd3a* and *RFT1* in *ossap5* was significantly lower than that in NIP at bright and dark points, excepting the *RFT1* expression had no remarkable changes at the dark point (**Figures 3D, F**).

**Figure 4:**
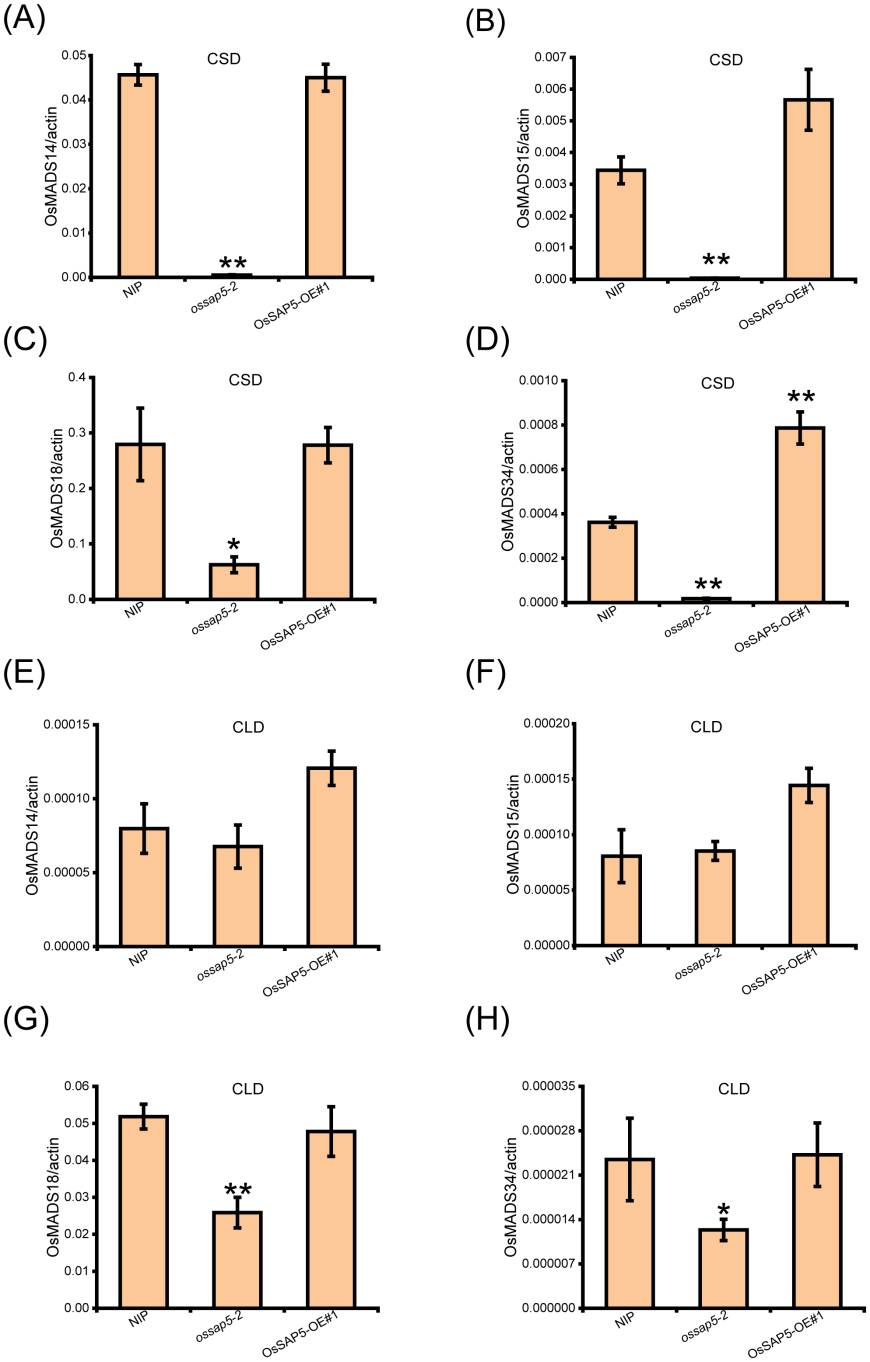
Expression analysis of *OsMADS* genes in the SAM of transgenic plants of *OsSAP5*. **(A–D)** Expression levels of *OsMADS14* **(A)**, *OsMADS15* **(B)**, *OsMADS18* **(C)**, and *OsMADS34* **(D)** in NIP, *ossap5-2*, OsSAP5-OE#1 under CSD condition. Values are presented as means ± SD (*n* = 3, *P < 0.05; **P < 0.01; Student’s *t*-test). **(E–H)**. Expression levels of *OsMADS14* **(E)**, *OsMADS15* **(F)**, *OsMADS18* **(G)**, and *OsMADS34* **(H)** in NIP, *ossap5-2*, OsSAP5-OE#1 under CLD condition. Values are presented as means ± SD (*n* = 3, *P < 0.05; **P < 0.01; Student’s *t*-test). Samples are prepared from SAM of 15 different plants of every transgenic line.

The *Hd3a* and *RFT1* are crucial factors for OsFD1 inducing MADS-box (*OsMADS14*, *OsMADS15*, *OsMADS18* and *OsMADS34*) genes expression, which regulate the rice heading date (Kobayashi et al., 2012). Therefore, the expressions of *MADS* genes were checked in the SAM of NIP and *OsSAP5* transgenic materials. The results showed that four *MADS* genes were reduced obviously in *ossap5-2*, but not significantly changed in OsSAP5-OE#1 under CSD condition (**Figures 4A–D**). Under CLD condition, the *OsMADS14* and *OsMADS15* expression did not differ markedly compared with NIP (**Figures 4E, F**). However, the *OsMADS18* and *OsMADS34* expression declined significantly in *ossap5-2* (**Figures 4G, H**). The expression change of these genes related to heading was consistent with the late-heading phenotype of *ossap5*-*CRISPR* mutants.

### *OsSAP5* expression pattern and subcellular localization

**Figure 5:**
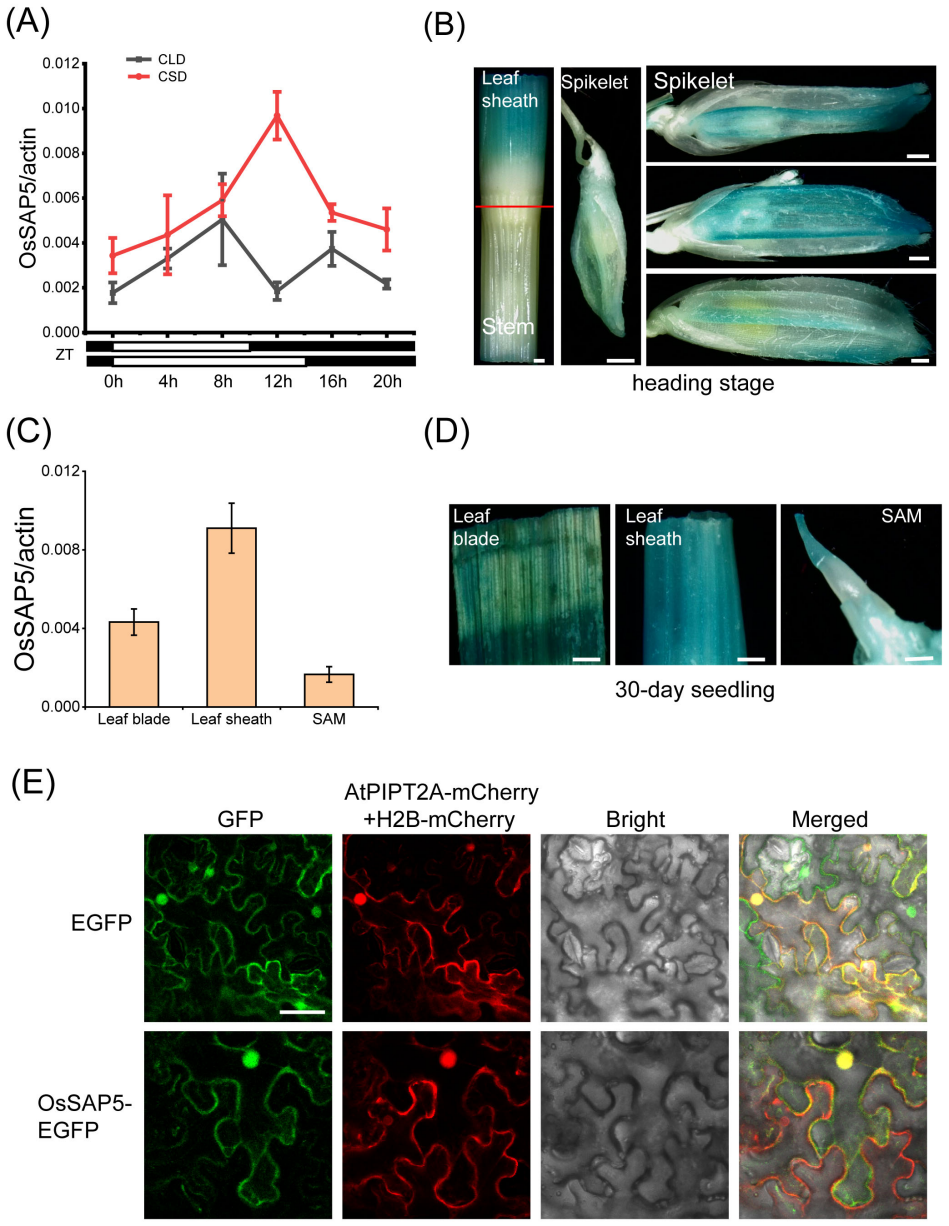
Expression pattern analysis of *OsSAP5*. **(A)** qRT-PCR analysis of the rhythmic expression of *OsSAP5*. Values are presented as means ± SD (*n* = 3). **(B)** Gus histochemical staining of *pOsSAP5::gus* transgenic lines in heading stage. Bar = 500 μm. **(C)** Expression pattern analysis of *OsSAP5* in different tissues of NIP. Values are presented as means ± SD (*n* = 3). **(D)** Gus histochemical staining of 30-day-old *pOsSAP5::gus* transgenic lines in leaf blade, leaf sheath, and shoot apical meristem (SAM). Bar = 300 μm. **(E)** Subcellular localization of the OsSAP5-EGFP fusion protein in tobacco leaf. AtPIPT2A-mCherry is used as a cytomembrane marker. H2B-mCherry is used as a nuclear marker. Free EGFP is used as the control. Bar = 50 μm.

To further understand the biological function of *OsSAP5*, we detected the spatiotemporal expression pattern of *OsSAP5* in different tissues. Expression analysis showed that *OsSAP5* exhibited rhythmic expression patterns under both CSD and CLD conditions (**Figure 5A**). *OsSAP5* was expressed in the leaf blade, leaf sheath and SAM of 30-day seedling (**Figure 5C**). Histochemical assays showed that the GUS activity was detected in the same tissue with qRT-PCR (**Figure 5D**). GUS staining assay also showed that *OsSAP5* was expressed in the spikelet with different sizes (**Figure 5B**). A transgenic material containing *pOsSAP5::OsSAP5-EGFP* was constructed for checking the expression pattern of *OsSAP5* as well. Fluorescence microscope observation assays showed that *OsSAP5* expressed in the phloem and xylem cells of vessel of the leaf sheath (**Supplementary Figure S3**). We co-expressed *OsSAP5*-*EGFP* with membrane-labeled *AtPIPT2A*-*mCherry* and nucleus-labeled *H2B*-*mCherry* in tobacco leaves. The results showed that the green overlapped with the red fluorescence in nucleus of tobacco cells, and not in the plasma membrane of tobacco cells (**Figure 5E**). It was speculated that OsSAP5 was localized in the cytoplasm and nucleus.

### OsSAP5 interacts with OsGF14c

**Figure 6:**
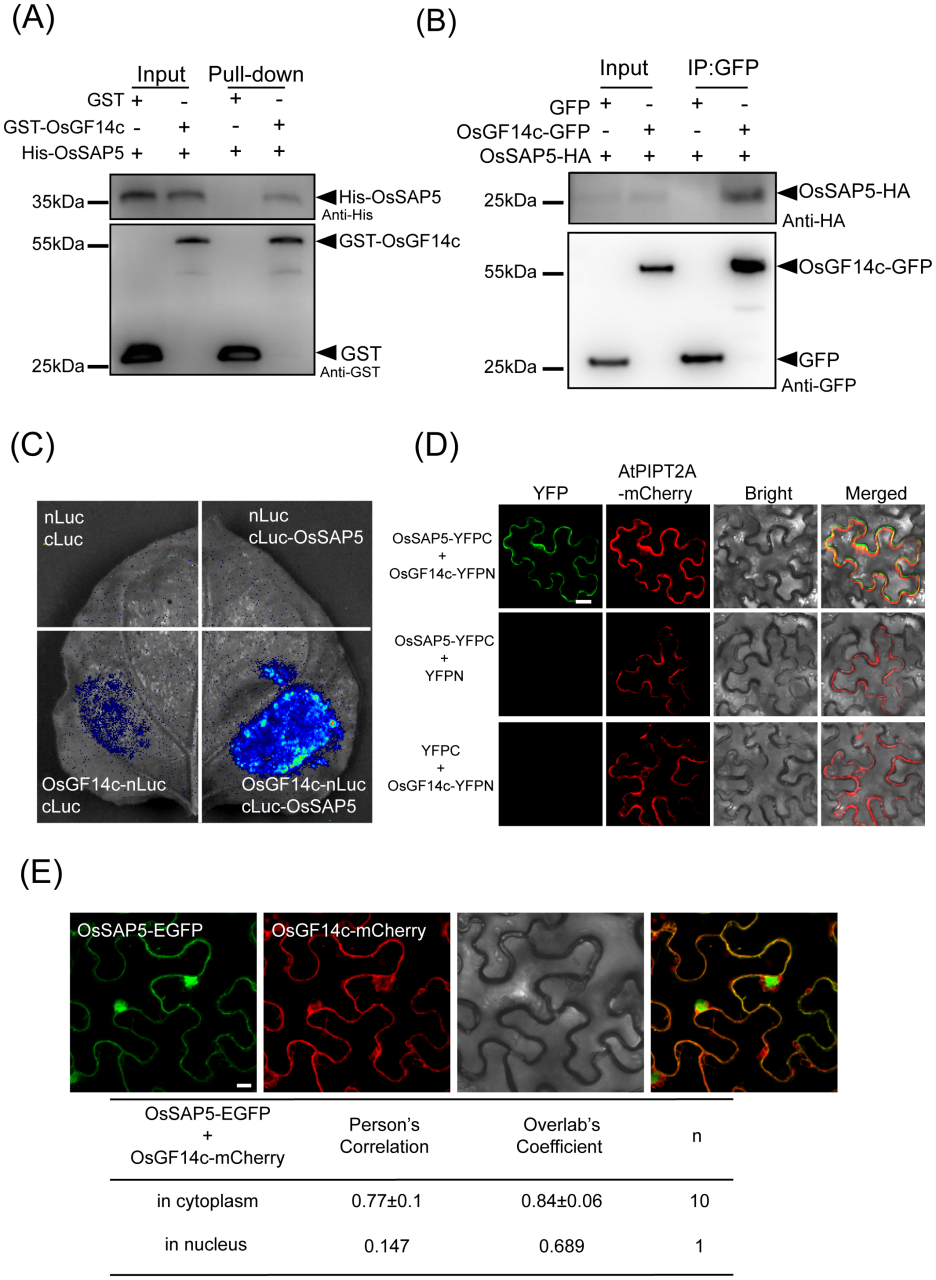
OsSAP5 interacts with OsGF14c. **(A)** An *in vitro* pull-down assay validates the interaction between OsSAP5 and OsGF14c. Induced His-OsSAP5 is incubated with the GST-OsGF14c or GST protein. Protein samples are immunoprecipitated with anti-GST beads and immunoblotted with anti-His and anti-GST antibodies. The symbols “-” and “+” represent the absence and presence of the corresponding proteins. **(B)** An *in vivo* Co-IP assay validates the interaction between OsSAP5 and OsGF14c in rice protoplasts. Proteins are isolated from protoplasts co-transfecting *p35s::OsGF14c-GFP* or *p35s::GFP* with *p35s::OsSAP5-HA*. Co-immunoprecipitation is performed using anti-GFP beads and immunoblotted with anti-HA and anti-GFP antibodies. **(C)** LCI assay verifies that OsSAP5 interacts with OsGF14c in the leaf epidermal cells of *N. benthamiana*. **(D)** BiFC assay shows that OsSAP5 interacts with OsGF14c in the leaf epidermal cells of *N. benthamiana* (*n* = 4 plants). Bar = 20 μm. **(E)** OsSAP5-GFP and OsGF14c-mCherry are co-localized in the leaf epidermal cells of *N. benthamiana*. Taking a statistic of fluorescence of GFP and mCherry in the cytoplasm (*n* = 10) and nucleus (*n* = 1) by Leica confocal microscopy. Bar = 10 μm.

To study the molecular mechanism of OsSAP5-mediated heading date, we performed a coimmunoprecipitation assay followed by liquid chromatography–tandem mass spectrometry (LC–MS/MS). The candidate protein, OsGF14c (LOC_Os08g33370), showed late-heading phenotype in its overexpression lines under SD condition (Purwestri et al., 2009; **Supplementary Table S2**). The pull-down assay showed that GST-OsGF14c was bound to His-OsSAP5, while no binding was detected between GST alone and His-OsSAP5 (**Figure 6A**). In addition, Co-IP assay indicated that OsGF14c interacted with OsSAP5 in the rice protoplasts (**Figure 6B**). The LCI assay also showed that co-transformation with OsGF14c-nLuc and cLuc-OsSAP5 displayed a considerable interaction signal in tobacco cells, but the controls did not (**Figure 6C**). The BiFC assay confirmed again that only the co-expression of OsGF14c and OsSAP5 could detect the YFP fluorescence signal in tobacco cells (**Figure 6D**). OsGF14c was localized in the cytoplasm (**Supplementary Figure S4**). The co-expression of OsGF14c-mCherry and OsSAP5-EGFP assay showed that the interaction of OsGF14c and OsSAP5 was only in the cytoplasm (**Figure 6E**), and the results were consistent with the localization of OsGF14c.

The 14-3–3 family proteins OsGF14b, OsGF14d, and OsGF14e were the homologous proteins of OsGF14c that regulated the heading date of rice (Taoka et al., 2011). The pull-down assay confirmed that OsSAP5 also interacts with OsGF14b, OsGF14d, and OsGF14e (**Supplementary Figure S5**). Taken together, OsSAP5 interacts with OsGF14c *in vivo* and *in vitro*.

### OsSAP5 has E3 ubiquitin ligase activity and may promote OsGF14c degradation

**Figure 7:**
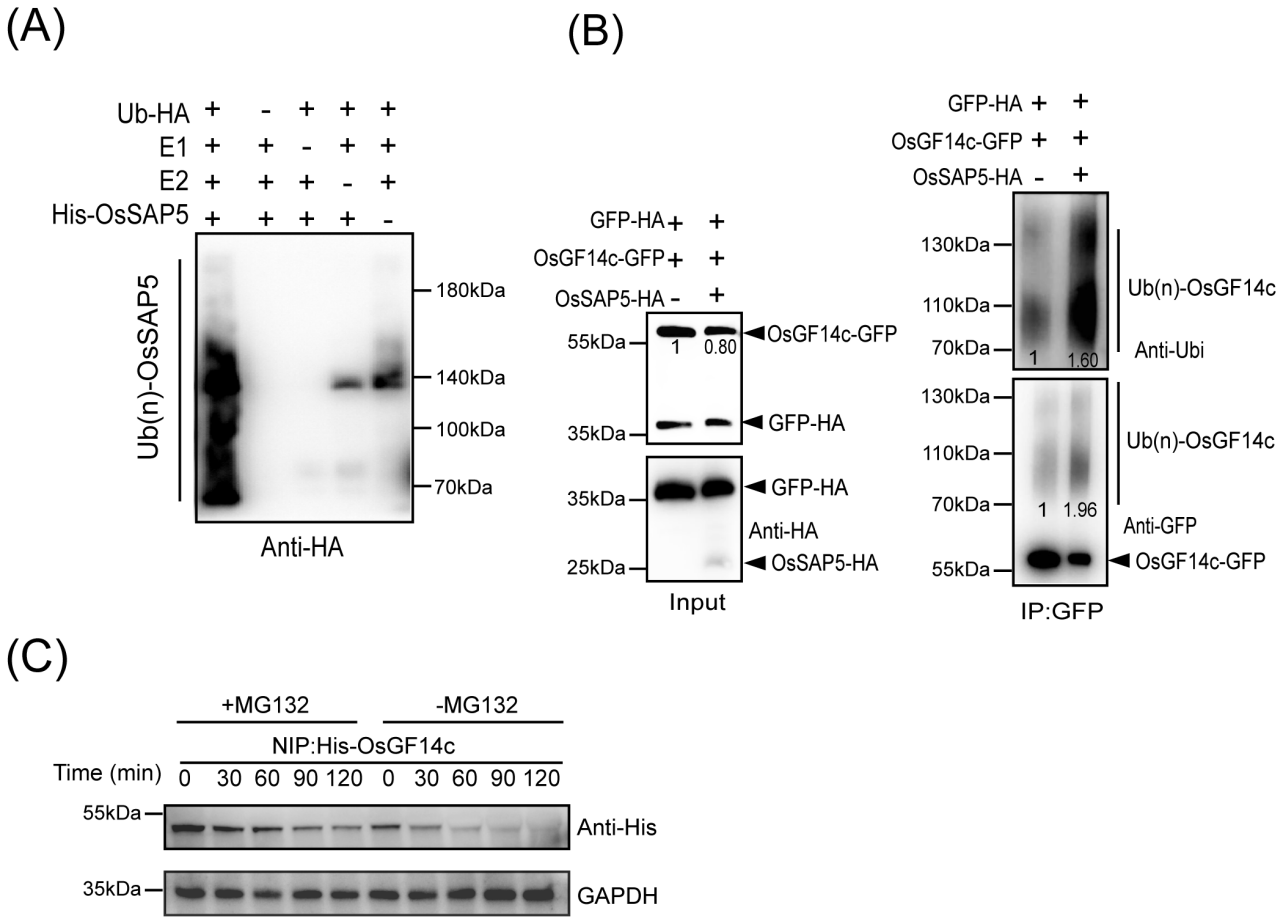
OsSAP5 ubiquitinates OsGF14c. **(A)** OsSAP5 is an E3 ubiquitin ligase. E1 (Ub-activating enzyme1, human), E2 (UbcH5b), HA-tagged Ub protein (Ub-HA), and His-OsSAP5 are added to reactions in the presence of ATP. Ubiquitin-bound protein of OsSAP5 is detected by immunoblotting with anti-Ub antibodies. His-OsSAP5 is 35.83 kDa. **(B)** OsSAP5 promotes the ubiquitination of OsGF14c *in vivo*. Proteins are isolated from protoplasts co-transfected with *p35s::OsGF14c-GFP* or *p35s::GFP-HA* with *p35s::OsSAP5-HA*. Co-immunoprecipitation is performed using anti-GFP beads and immunoblotted with anti-HA, anti-ubi, and anti-GFP antibodies. In the input results, the ratio of immunoblot values of OsGF14c-GFP and GFP-HA was set as 1. In co-immunoprecipitation results, the band of left was set as 1. **(C)** OsGF14c is degraded through the 26S proteasome pathway. Purified His-OsGF14c is incubated with equal amounts of total proteins extracted from NIP plant leaf at the indicated times at 25°C. +MG132 means 300 μm MG132 is added to NIP samples to examine protein degradation through the 26S proteasome pathway. –MG132 means DMSO is added to NIP samples. His-OsGF14c is immunoblotted with anti-His antibodies. Loading of protein is shown by the GAPDH antibodies.

OsSAP5 contains two zinc-finger domains, A20 and AN1. A20 and AN1 were reported to have the E3 ubiquitin ligase activity (Heyninck et al., 1999). Therefore, the purified His-OsSAP5 protein was incubated with E1, E2, and ubiquitin-HA (Ub-HA) proteins in an *in vitro* ubiquitination assay, and the results were analyzed by immunoblotting using anti-HA antibodies. A series of molecular masses was detected only when all reaction components were added, whereas lacking any one of the components E1, E2 Ub-HA, or His-OsSAP5 failed to produce a positive result (**Figure 7A**), which indicated that OsSAP5 was an E3 ubiquitin ligase. The co-transformation of OsSAP5-HA, OsGF14c-GFP, and GFP-HA or OsGF14c-GFP and GFP-HA into rice protoplast was carried out, and the results showed that OsGF14c-GFP was more degraded and ubiquitinated when OsSAP5-HA was expressed together (**Figure 7B**). Substrate ubiquitination modification had different polyubiquitin chains, and lysine-48 (K48)-linked chains were the signal for 26S proteasomes (Al-Saharin et al., 2022). Thus, the OsGF14c level in the presence or absence of MG132, a chemical inhibitor of the 26S proteasome, was examined. Immunoblot analysis showed that OsGF14c abundance decreased slowly in the presence of MG132 but decreased quickly in the absence of MG132 (**Figure 7C**). Collectively, we confirmed that OsSAP5 is an E3 ubiquitin ligase and may promote the ubiquitination of OsGF14c.

### OsGF14c negatively regulates heading date by reducing the *Hd3a* and *RFT1* expression

**Figure 8:**
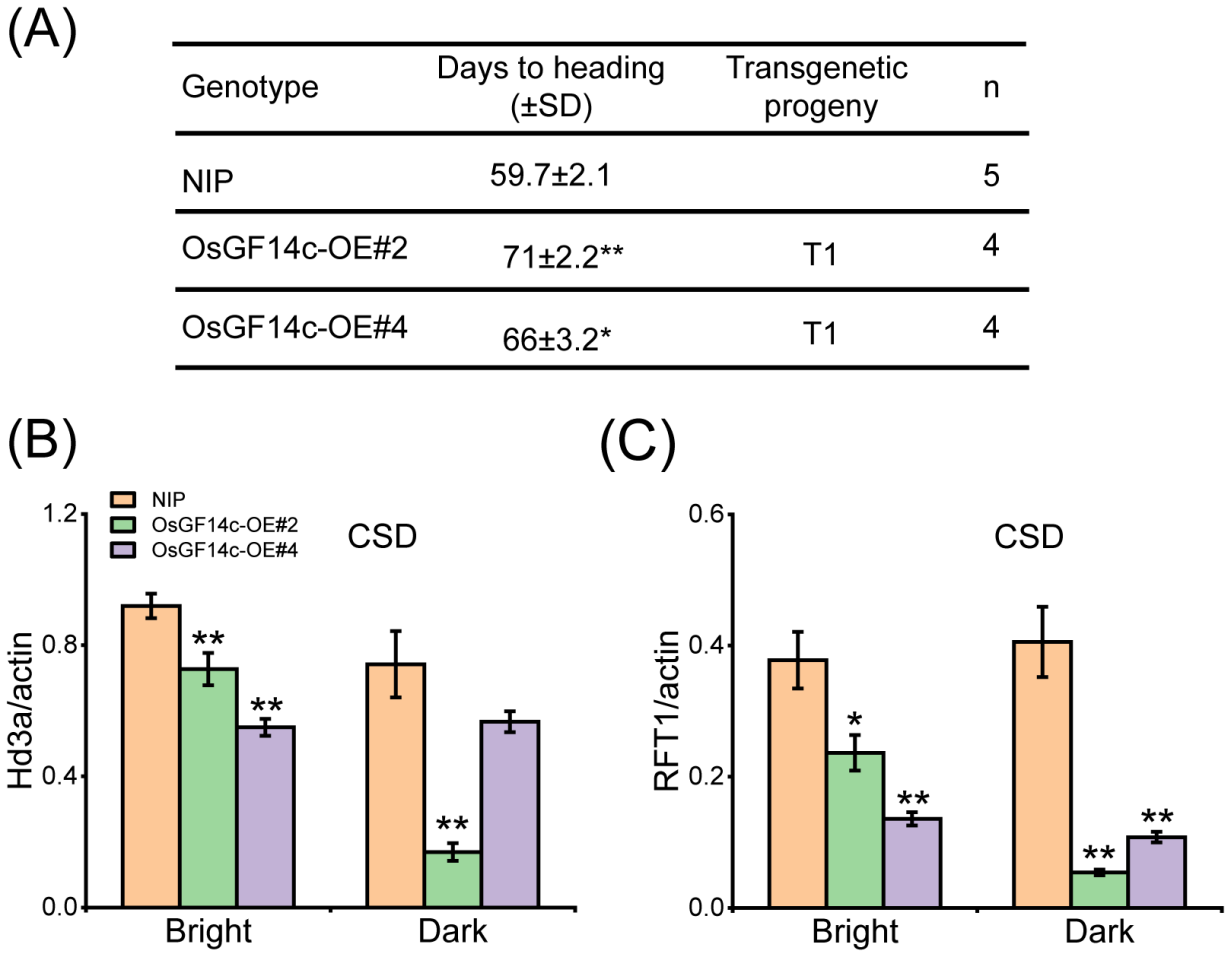
Heading date of *OsGF14c* overexpression transgenic material under CSD condition. **(A)** Days to heading of NIP, OsGF14c-OE#2, and OsGF14c-OE#4 under CSD condition. Values are presented as means ± SD (*P < 0.05; **P < 0.01; Student’s *t*-test). **(B, C)** The expression of *Hd3a* **(B)** and *RFT1* **(C)** in NIP, OsGF14c-OE#2, and OsGF14c-OE#4 under CSD condition. Values are presented as means ± SD (*n* = 3, *P < 0.05; **P < 0.01; Student’s *t*-test).

*OsGF14c*-overexpression lines were later than NIP in heading date (Purwestri et al., 2009). However, the detailed reason is not understood. The two CRISPR mutants, *osgf14c-1* and *osgf14c-2*, were constructed by the CRISPR/Cas9 genome editing approach. The *osgf14c-1* showed the deletion of 55 bases in *OsGF14c* coding sequence, and the *osgf14c-2* lost one base and led to a premature stop codon (**Supplementary Figure S6D**). Two CRISPR-mutants did not have extremely different changes in heading date compared to NIP (**Supplementary Figures S6A–C**). The qRT-PCR and immunoblotting with anti-MYC assays showed that the two overexpressing lines of OsGF14c (OsGF14c-OE#2 and OsGF14c-OE#4) were constructed successfully (**Supplementary Figures S7A, B**). OsGF14c-OE#2 and OsGF14c-OE#4 showed the delayed heading date for about 11 days and 6 days compared to NIP under CSD condition (**Figure 8A**). The expression of *Hd3a* and *RFT1* declined extremely in the OsGF14c-OE#2 than in NIP under bright and dark conditions (**Figures 8B, C**). OsGF14c-OE#4 also showed a significant decrease of *Hd3a* and *RFT1* expression compared to NIP, except the *Hd3a* expression under dark condition (**Figures 8B, C**). These results were consistent with the results of *ossap5*-*CRISPR* mutants influencing the heading date by reducing *Hd3a* and *RFT1* expression.

## Discussion

### OsSAP5 positively regulates heading date in rice

This study firstly reported that SAP family protein was involved in the reproductive growth of rice. In *Arabidopsis*, only *AtSAP9* was reported to have the function in flowering time (Kang et al., 2017). In this study, the *ossap5*-*CRISPR* mutants delayed the heading date under NSD, CSD, and CLD conditions (**Figures 1A, B**, **3A, B**). However, the mutants had no changes in heading date under NLD, the reason of that may be that *OsSAP5* was associated with temperature in the wild field. SAP family proteins were also involved in biotic and abiotic stresses (Mukhopadhyay et al., 2004; Kang et al., 2011, 2017). The *ossap5*-*CRISPR* mutants showed weak tolerance to heat stress in the seedling, but the molecular mechanism needed to be further explored.

A cotton protein, GaZnF, contains an A20 domain and had been reported to bind to a MYB-box element present in the *GUSP1* (cotton stress responsive gene) promoter, suggesting a possible transcription regulator activity of SAP protein (Zahur et al., 2012). Thus, OsSAP5 transcriptional activity in rice protoplasts was performed by dual luciferase reporter (DLR) system, and VP16 was used as the transcriptional activator (**Supplementary Figure S8**). OsSAP5 expression resulted in a similar firefly luciferase/*Renilla* luciferase (LUC/REN) activity as did the empty GAL4BA vector (**Supplementary Figure S8**). The results indicated that OsSAP5 did not have transcriptional activity. OsSAP5 could not regulate the florigen *Hd3a* and *RFT1* expression directly either, although *Hd3a* and *RFT1* showed a remarkable decline in *ossap5*-*CRISPR* mutants (**Figures 3C–F**).

### OsGF14c overexpression has a delay in heading date

Overexpression of *Hd3a* or *OsFD1* promoted heading date in rice (Kojima et al., 2002; Peng et al., 2021); however, *OsGF14c*-overexpressed plants delayed heading date by approximately 15 days under SD condition (Purwestri et al., 2009). This phenotype of genetic material shows that FAC components may be balanced for FAC activity. The expressions of *OsMADS* genes could be induced by effector Hd3a and FD1 together in the rice protoplast system (Cai et al., 2021). *OsGF14c* had a higher transcription level than *Hd3a* in rice tissue (**Supplementary Figure S9A**). Therefore, the effector of p35s::Hd3a, pGF14c::OsFD1, and pHd3a::OsGF14c was compared with those of p35s::Hd3a, pGF14c::OsFD1, and pGF14c::OsGF14c in rice protoplasts. The results revealed no significant changes in *OsMADS* expression in response to altered OsGF14c protein levels (**Supplementary Figure S9B**). It may be identified that FAC components do not have a balance for each other.

FTL12 interacted with OsGF14b and OsFD1 to form the florigen repression complex (FRC) by competing with Hd3a for binding OsGF14b (Zheng et al., 2023), but Hd3a could not compete with FTL12 for binding OsGF14b (Zheng et al., 2023). Thus, OsGF14c was more easily interacted with FTL12 to form more FRC than FAC. This may be another possible reason for the OsGF14c-overexpressed lines prohibiting heading date. OsFTL4 was also reported to compete with Hd3a interacting with 14-3–3 and negatively regulates the heading date (Gu et al., 2022). HBF1/HBF2, two paralogs with OsFD1, are mainly expressed in rice leaves (Brambilla et al., 2017). HBFs influenced the *Hd3a*/*RFT1* expression by regulating *Ehd1* directly and also interacted with Hd3a directly, with RFT1 by OsGF14c as the bridge (Brambilla et al., 2017). In our study, *Hd3a* and *RFT1* expression was lower in OsGF14c-overexpression than NIP under CSD condition (**Figures 8B, C**). Thus, HBFs may be another reason to explain why *OsGF14c* overexpression has a late-heading phenotype. How the *OsGF14c*-overexpression lines play in heading under long day conditions needs further study.

### OsSAP5 has E3 ubiquitin activity and interacts with OsGF14c

OsSAP5 had E3 ubiquitin ligase activity in our study (**Figure 7A**). A lot of SAP proteins have been reported to have E3 ubiquitin ligase activity in wheat and *Arabidopsis*, such as *TaSAP5*, *AtSAP5*, *AtSAP9*, and so on (Choi et al., 2012; Kang et al., 2017; Zhang et al., 2017).

We got the OsGF14c protein by IP-MS and confirmed that OsSAP5 interacts with OsGF14c (**Figure 6**). *OsGF14c*-overexpression plants and *ossap5*-*CRISPR* mutants showed a similar heading phenotype in rice under short day condition (**Figures 1B**, **8A**). OsGF14c was degraded by UPS system (**Figure 7C**). OsSAP5 may promote the ubiquitination of OsGF14c (**Figure 7B**). Thus, OsSAP5 may positively regulate heading date by promoting the degradation of OsGF14c ubiquitination in rice. The *OsGF14c*-*CRISPR* mutants showed a comparable phenotype in heading date compared to NIP. The reason may be that OsGF14b, OsGF14d, and OsGF14e have functional redundancy in regulating the heading date. That might also be the reason why *OsSAP5*-overexpression lines showed the similar heading date with NIP, as 14-3–3 protein could not be degraded by OsSAP5 completely.

## Conclusion

**Figure 9:**
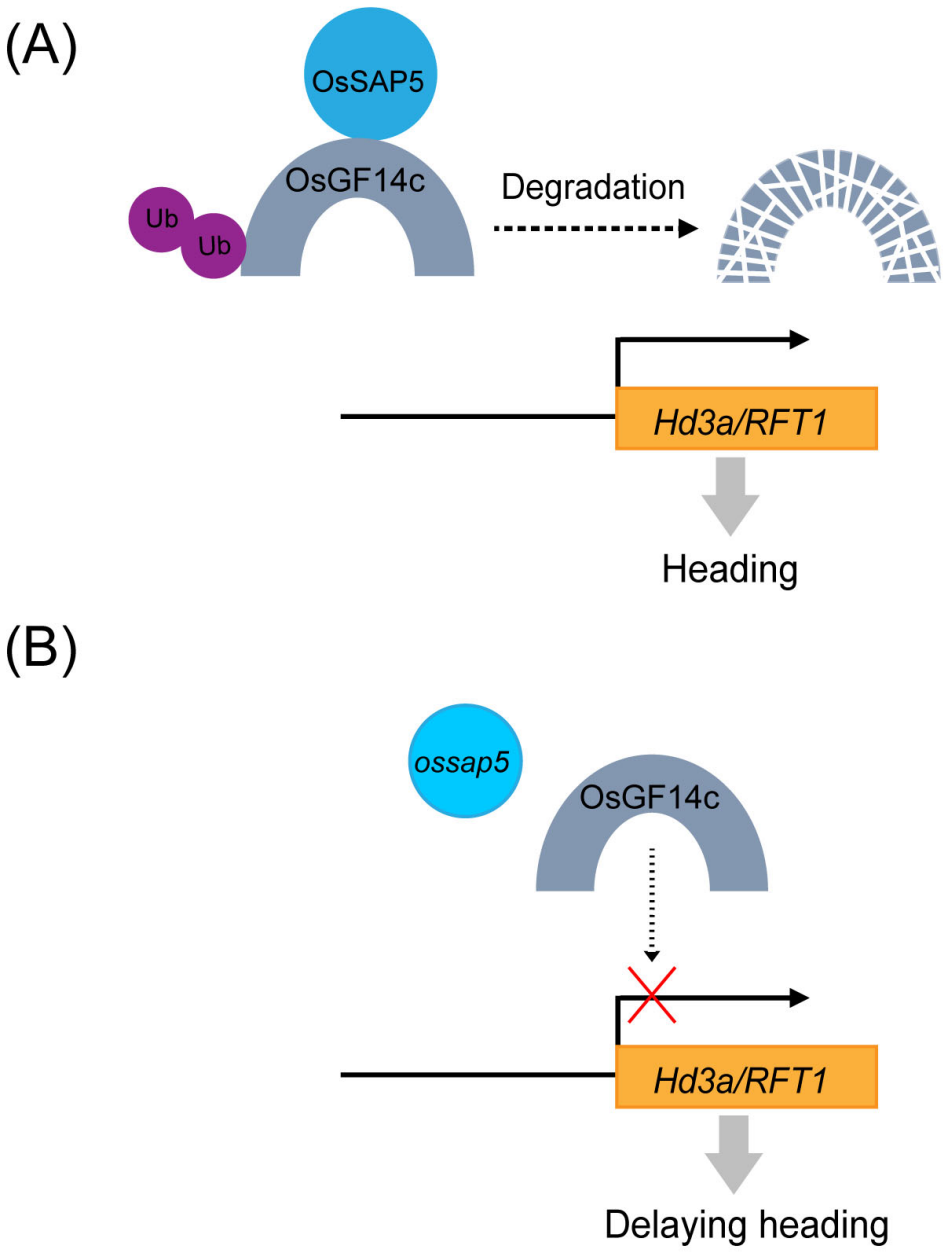
A proposed working model for the regulation of rice heading by OsSAP5. **(A)** In wild type, OsSAP5 may promote OsGF14c ubiquitination and OsGF14c may be degraded by 26S proteasome, and *Hd3a*/*RFT1* is normally expressed. **(B)** In the *ossap5* mutant, more OsGF14c decrease the expression of *Hd3a*/*RFT1*, which then delays heading date. UPS, ubiquitin/26S proteasome system.

The study first demonstrates that *ossap5*-*CRISPR* mutants delay the heading date by reducing the *Hd3a* and *RFT1* expression. Similarly, the *OsGF14c*-overexpression displayed comparable phenotypic characteristics with delayed heading and downregulation of *Hd3a/RFT1* expression. Further molecular characterization revealed that OsSAP5 functions as an E3 ubiquitin ligase that may facilitate the ubiquitination of OsGF14c. Thus, OsSAP5 may mediate the proteasomal degradation of OsGF14c through the 26S proteasome pathway. A schematic model summarizing the regulatory relationship between OsSAP5 and OsGF14c in controlling rice heading date is presented in **Figure 9**.

## Data Availability

The original contributions presented in the study are included in the article/**Supplementary Material**, further inquiries can be directed to the corresponding author/s.

## Supplementary material

Supplementary Figure 1 Electrophoresis diagram of *HYG* in *OsSAP5* overexpression lines. The positive band is 481 bp; the primer is HYG-F/HYG-R.

Supplementary Figure 2 Rhythmic expression of heading-related genes in NIP, *ossap5-2*, and OsSAP5-OE#1 under CSD condition. RNA is extracted from 15-day seedling. ZT is the means of Zeitgeber time.

Supplementary Figure 3 Microscope observation of *pOsSAP5::OsSAP5-EGFP*. Microscope images of *pOsSAP5::OsSAP5-EGFP* transgenic plants. Bar = 50 μm. GFP fluorescence observed in the cell of the phloem (Phl) and xylem (Xy).

Supplementary Figure 4 Subcellular localization of the OsGF14c protein. Subcellular localization of the OsGF14c-EGFP in tobacco leaf. AtPIPT2A-mCherry is used as a plasma membrane marker. H2B-mCherry is used as a nuclear marker. The free EGFP is used as the control. Bar = 50 μm.

Supplementary Figure 5 OsSAP5 interacts with OsGF14b, OsGF14d, and OsGF14e. An *in vitro* pull-down assay validates the interaction between OsSAP5 and OsGF14b, d, e. The induced His-OsSAP5 is incubated with the GST-OsGF14b or GST-OsGF14d or GST-OsGF14e or GST protein. Protein samples are immunoprecipitated with anti-GST beads and immunoblotted with anti-His and anti-GST antibodies. The symbols “-” and “+” represent the absence and presence of the corresponding proteins.

Supplementary Figure 6 Heading date of *OsGF14c* mutants. **(A)** Heading phenotype of NIP and *OsGF14c* mutants under NSD condition. Bar = 10 cm; *osgf14c-1* and *osgf14c-2* represent two *OsGF14c* mutants. The rectangular box indicates rice heading. **(B)** Days to heading of NIP, *OsGF14c* mutants under NSD and NLD conditions. Values are presented as means ± SD (*n* = 15). **(C)** Days to heading of NIP, *OsGF14c* mutants under CSD and CLD conditions. Values are presented as means ± SD (*n* = 13). **(D)** Generation and identification of two *OsGF14c* mutants generated by CRISPR/Cas9 genome-editing approach. The target 1 site and protospacer adjacent motif (PAM) are indicated. The black dash indicates the deletion of a base pair.

Supplementary Figure 7 Construction of *OsGF14c* overexpression lines. **(A)** MYC-tag immunoprecipitate of *OsGF14c* overexpression plants. A total of 10 protein samples are detected with anti-MYC antibodies with loading shown by Ponceau S staining for RUBISCO. **(B)** The qRT-PCR analysis of the expression levels of *OsGF14c* in NIP and *OsGF14c* overexpression lines. Values are presented as means ± SD (*n* = 3, **P* < 0.05; ***P* < 0.01; Student’s *t*-test). OsGF14c-OE#2 and OsGF14c-OE#4 indicate two *OsGF14c* overexpression lines.

Supplementary Figure 8 Transcriptional activity analysis of *OsSAP5* using the DLR (dual-luciferase reporter) assay system. **(A)** Schematic representation of reporter and effectors. **(B)** Measurement of relative luciferase activity (LUC/REN). Values are presented as means ± SD (*n* = 3).

Supplementary Figure 9 The protein level of OsGF14c does not influence the function of FAC. **(A)** Expression pattern of *Hd3a* and *OsGF14c* in leaf blade, leaf sheath, and SAM. **(B)** The levels of *OsMADS14*, *OsMADS15*, and *OsMADS18* are detected by qRT-PCR. Values are presented as means ± SD (*n* = 3). The different protein levels of OsGF14c are supported by *pOsGF14c::OsGF14c* and *pHd3a::OsGF14c* in rice protoplasts.

Supplementary Table 1 Agronomic traits of NIP, *OsSAP5* mutants, and overexpression lines under NSD condition.
