# Correction: Stress-associated protein OsSAP5 regulates rice heading date through interacting with OsGF14c in rice

**DOI:** 10.3389/fpls.2025.1712287

**Published:** 2025-10-15

**Authors:** Xiaobo Zhu, Min Chen, Zhang Dong, Yihan Liu, Qingshan Mu, Farwa Basit, Jiaxin Liu, Jin Hu, Yajing Guan

**Affiliations:** ^1^ Hainan Institute, Zhejiang University, Sanya, China; ^2^ Institute of Crop Science, Zhejiang Key Laboratory of Crop Germplasm Innovation and Utilization, College of Agriculture and Biotechnology, Zhejiang University, Hangzhou, China; ^3^ Department of Biology, College of Science and Technology, Wenzhou-Kean University, Wenzhou, China

**Keywords:** rice (*Oryza sativa*), OsSAP5, heading date, OsGF14C, E3 ubiquitination ligase

The order of affiliations has been updated. Affiliation “Hainan Institute, Zhejiang University, Sanya, China” should be the first. Affiliation “Institute of Crop Science, Zhejiang Key Laboratory of Crop Germplasm Innovation and Utilization, College of Agriculture and Biotechnology, Zhejiang University, Hangzhou, China” should be the second.

An incorrect **Funding** statement was provided. The “Hainan Provincial Natural Science Foundation” should be “Hainan Provincial Natural Science Foundation of China”. The correct **Funding** statement reads:

“The author(s) declare financial support was received for the research and/or publication of this article. This research was supported by the Hainan Provincial Natural Science Foundation of China (322CXTD522), the Natural Science Foundation of Zhejiang Province (LQ24C130003), the National Natural Science Foundation of China (32072127), the PhD Scientific Research and Innovation Foundation of Sanya Yazhou Bay Science and Technology City (HSPHDSRF-2023-04-017) and Collaborative Innovation Center for Modern Crop Production co-sponsored by Province and Ministry (CIC-MCP)”.

The original version of this article has been updated.

