# Supplementary figures and images for: Stress-associated protein OsSAP5 regulates rice heading date through interacting with OsGF14c in rice
Source: Front Plant Sci. 2025 Sep 9;16:1589989. doi: 10.3389/fpls.2025.1589989 (PMC12454907; doi:10.3389/fpls.2025.1589989)

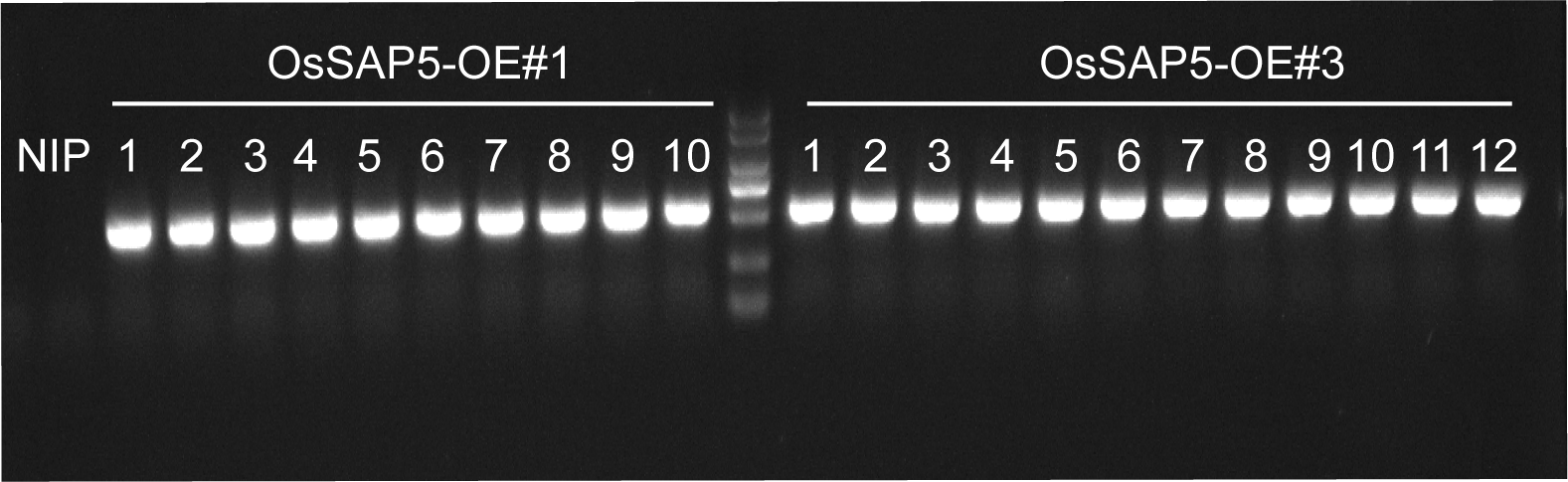

Supplement: Supplementary Figure 1 — Electrophoresis diagram of HYG in OsSAP5 overexpression lines. The positive band is 481 bp; the primer is HYG-F/HYG-R. [file DataSheet1.zip › Supplementary Figure S1-AI.tif]

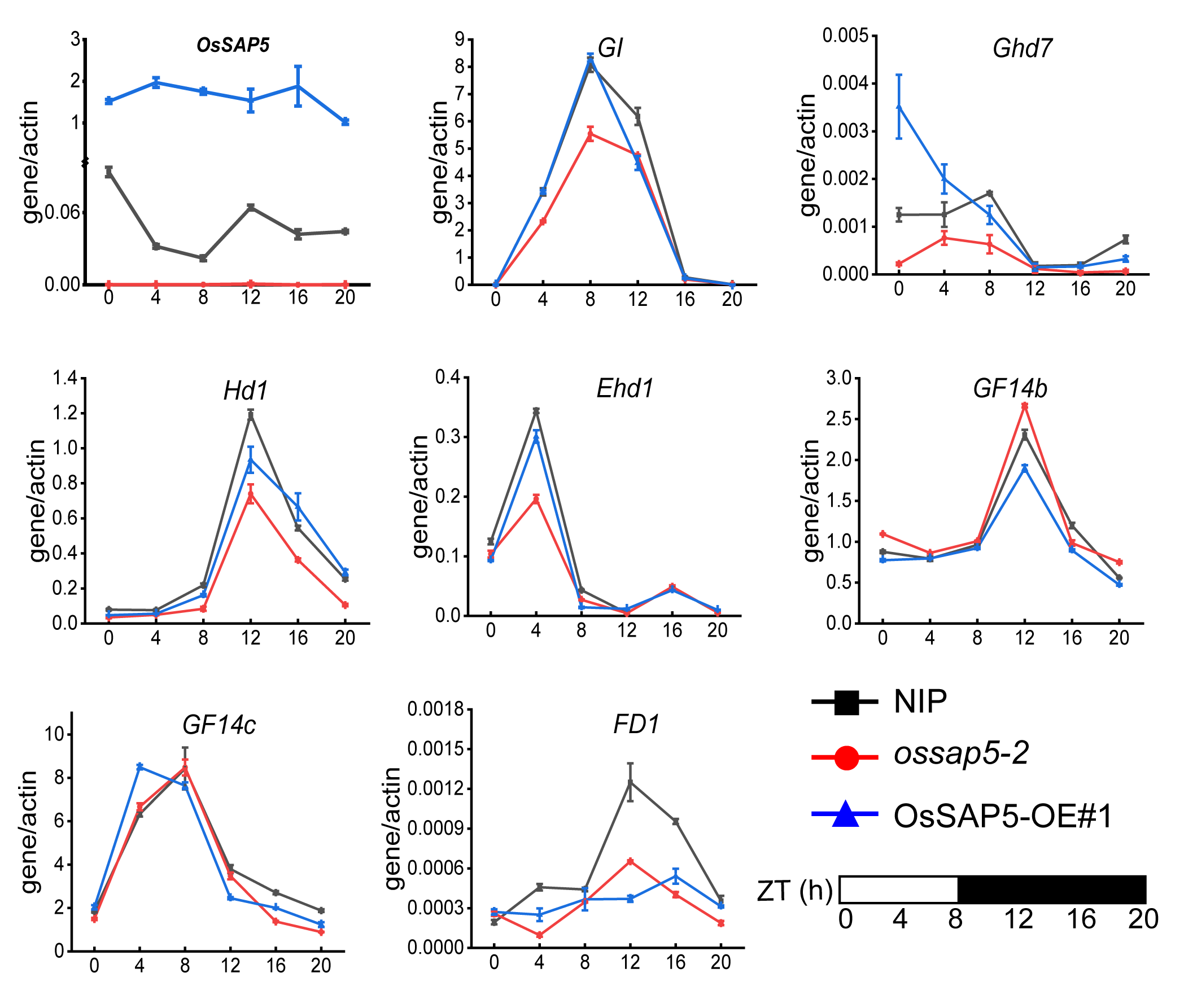

Supplement: Supplementary Figure 1 — Electrophoresis diagram of HYG in OsSAP5 overexpression lines. The positive band is 481 bp; the primer is HYG-F/HYG-R. [file DataSheet1.zip › Supplementary Figure S2.tif]

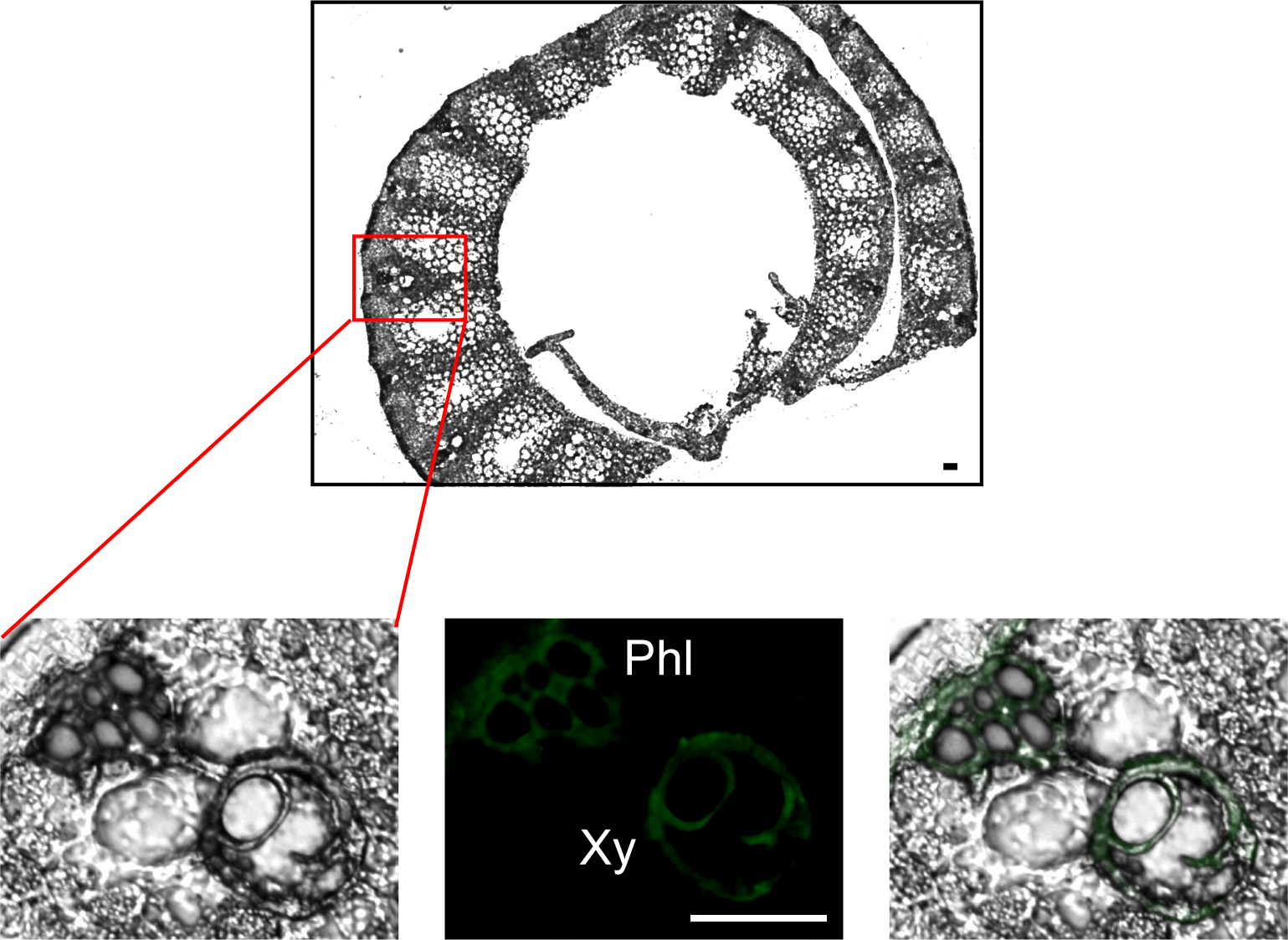

Supplement: Supplementary Figure 1 — Electrophoresis diagram of HYG in OsSAP5 overexpression lines. The positive band is 481 bp; the primer is HYG-F/HYG-R. [file DataSheet1.zip › Supplementary Figure S3.tif]

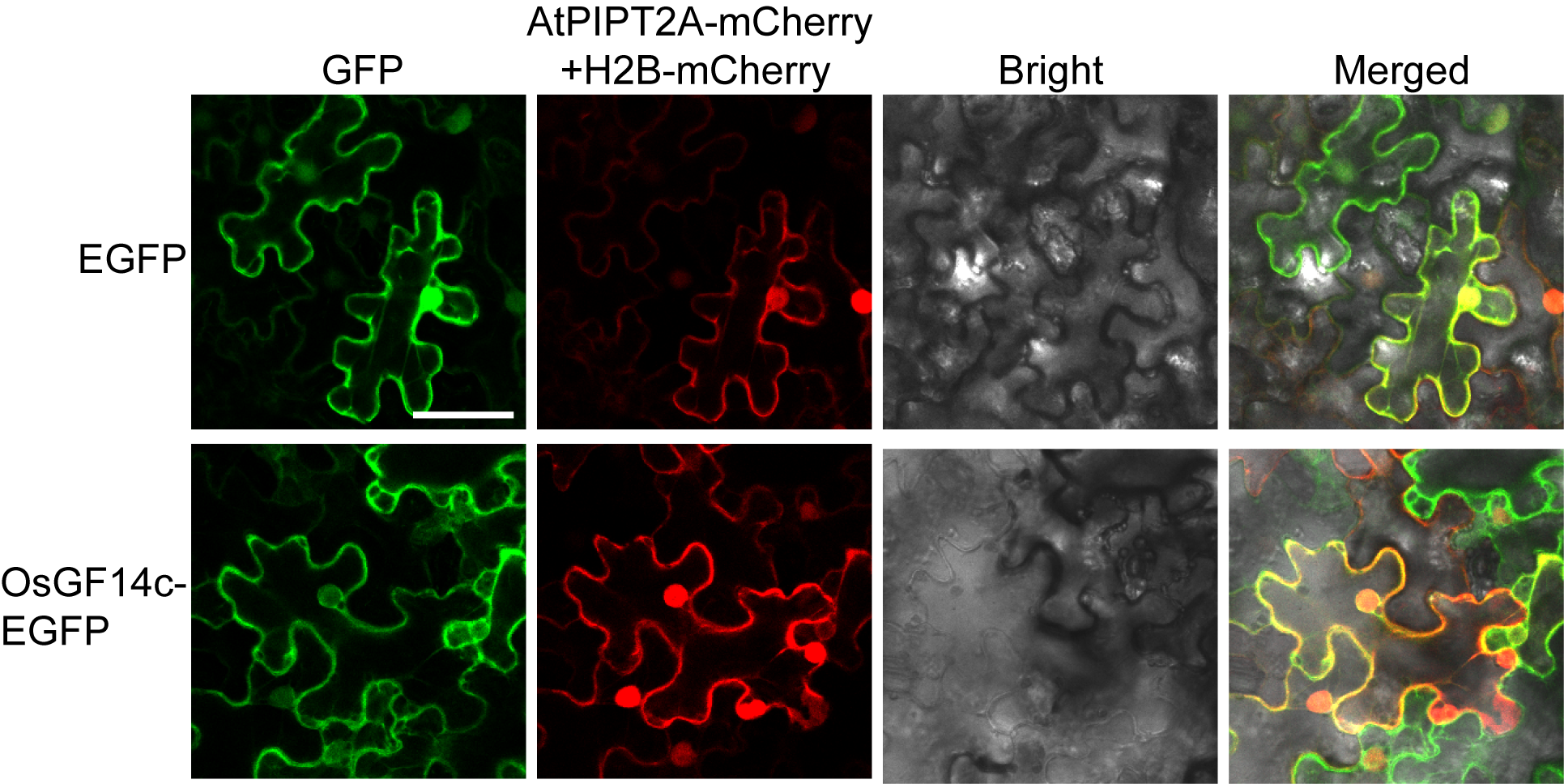

Supplement: Supplementary Figure 1 — Electrophoresis diagram of HYG in OsSAP5 overexpression lines. The positive band is 481 bp; the primer is HYG-F/HYG-R. [file DataSheet1.zip › Supplementary Figure S4.tif]

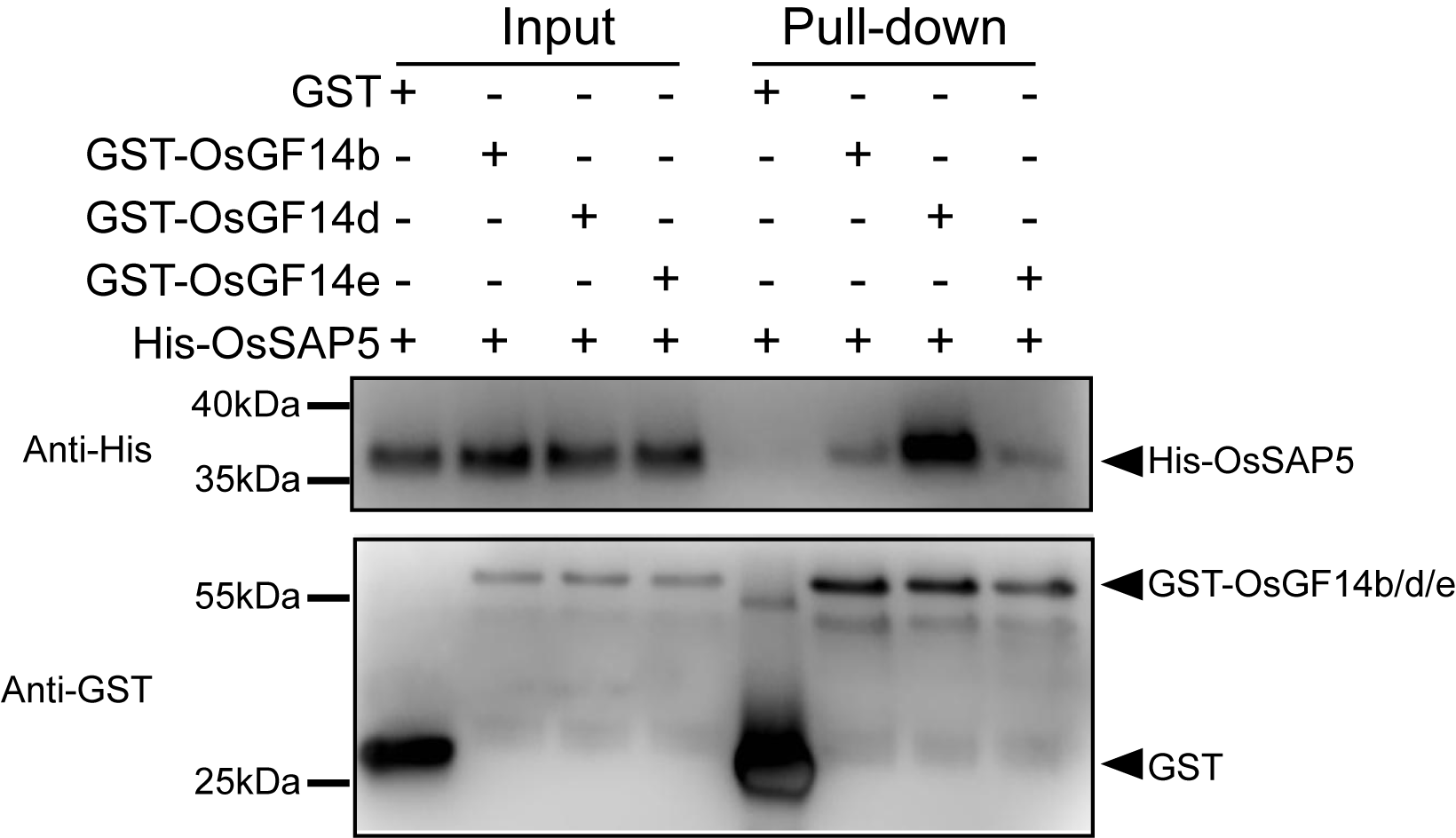

Supplement: Supplementary Figure 1 — Electrophoresis diagram of HYG in OsSAP5 overexpression lines. The positive band is 481 bp; the primer is HYG-F/HYG-R. [file DataSheet1.zip › Supplementary Figure S5-reviseion.tif]

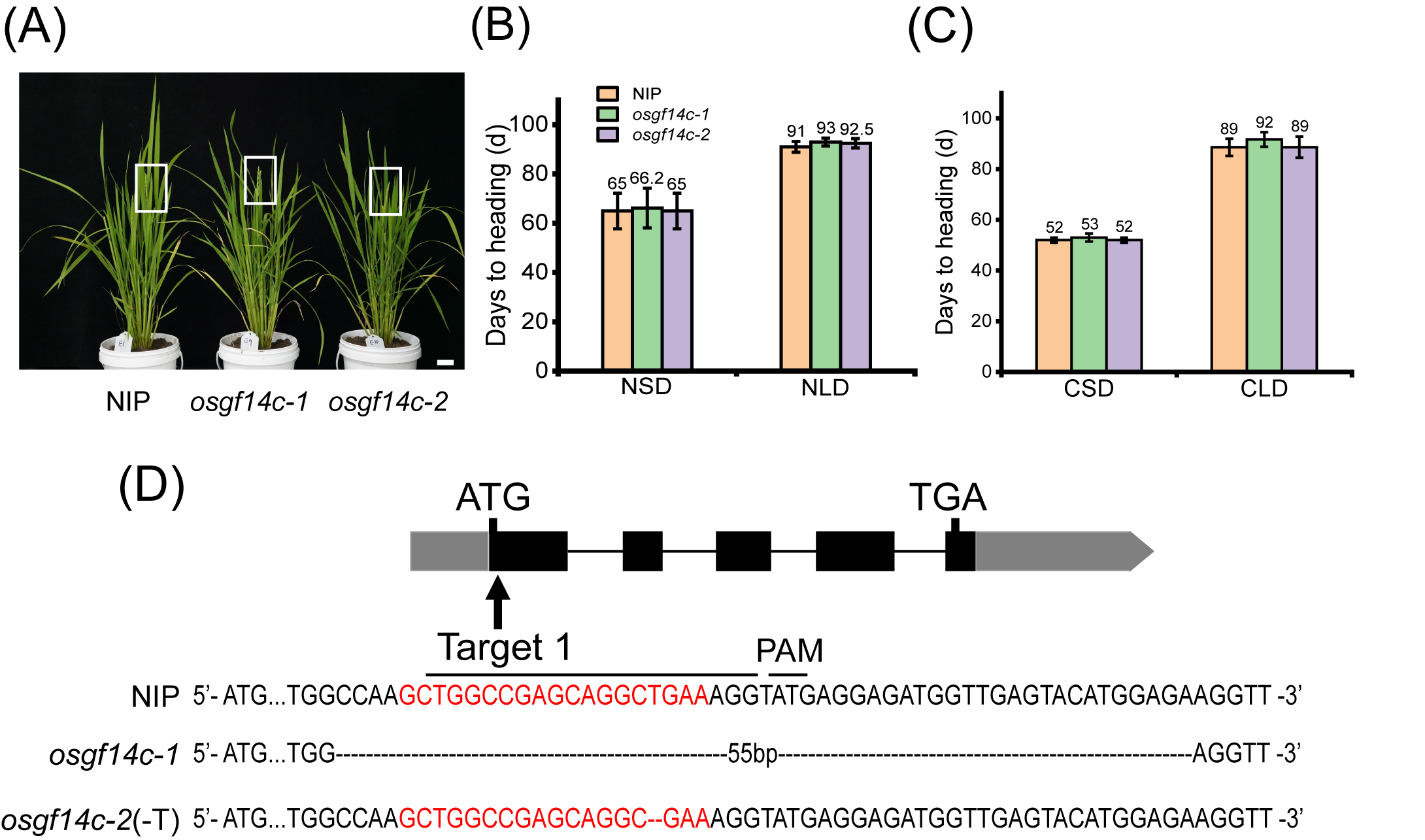

Supplement: Supplementary Figure 1 — Electrophoresis diagram of HYG in OsSAP5 overexpression lines. The positive band is 481 bp; the primer is HYG-F/HYG-R. [file DataSheet1.zip › Supplementary Figure S6.tif]

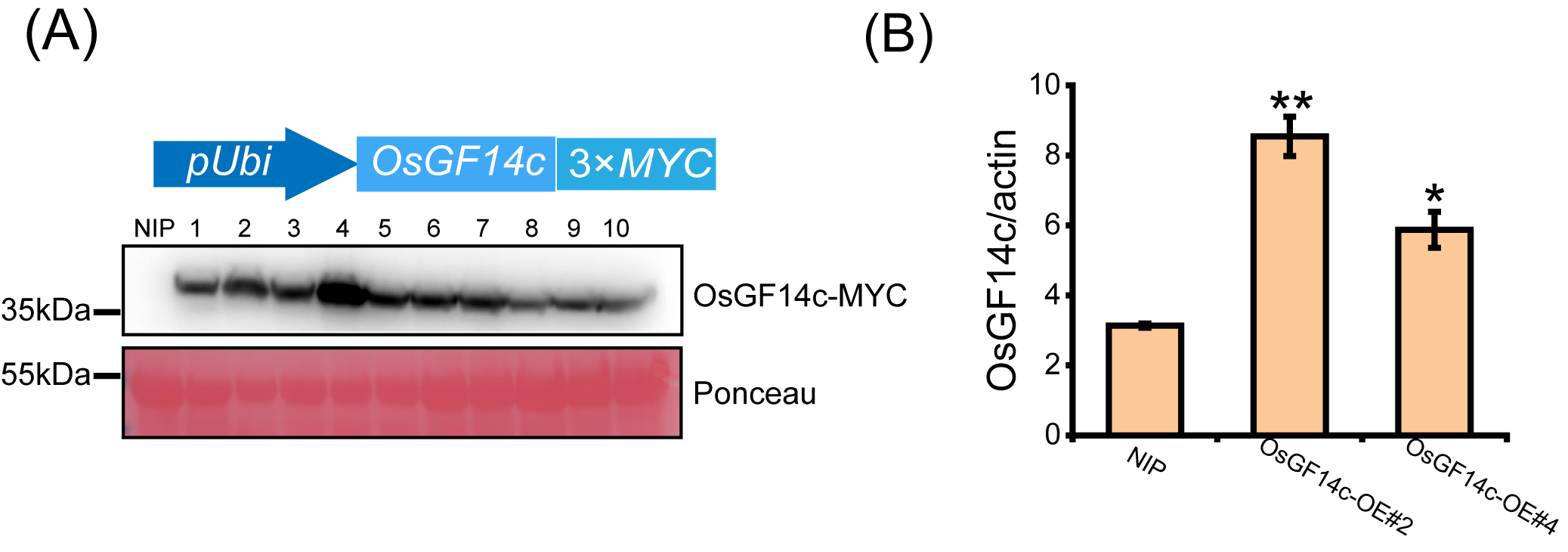

Supplement: Supplementary Figure 1 — Electrophoresis diagram of HYG in OsSAP5 overexpression lines. The positive band is 481 bp; the primer is HYG-F/HYG-R. [file DataSheet1.zip › Supplementary Figure S7.tif]

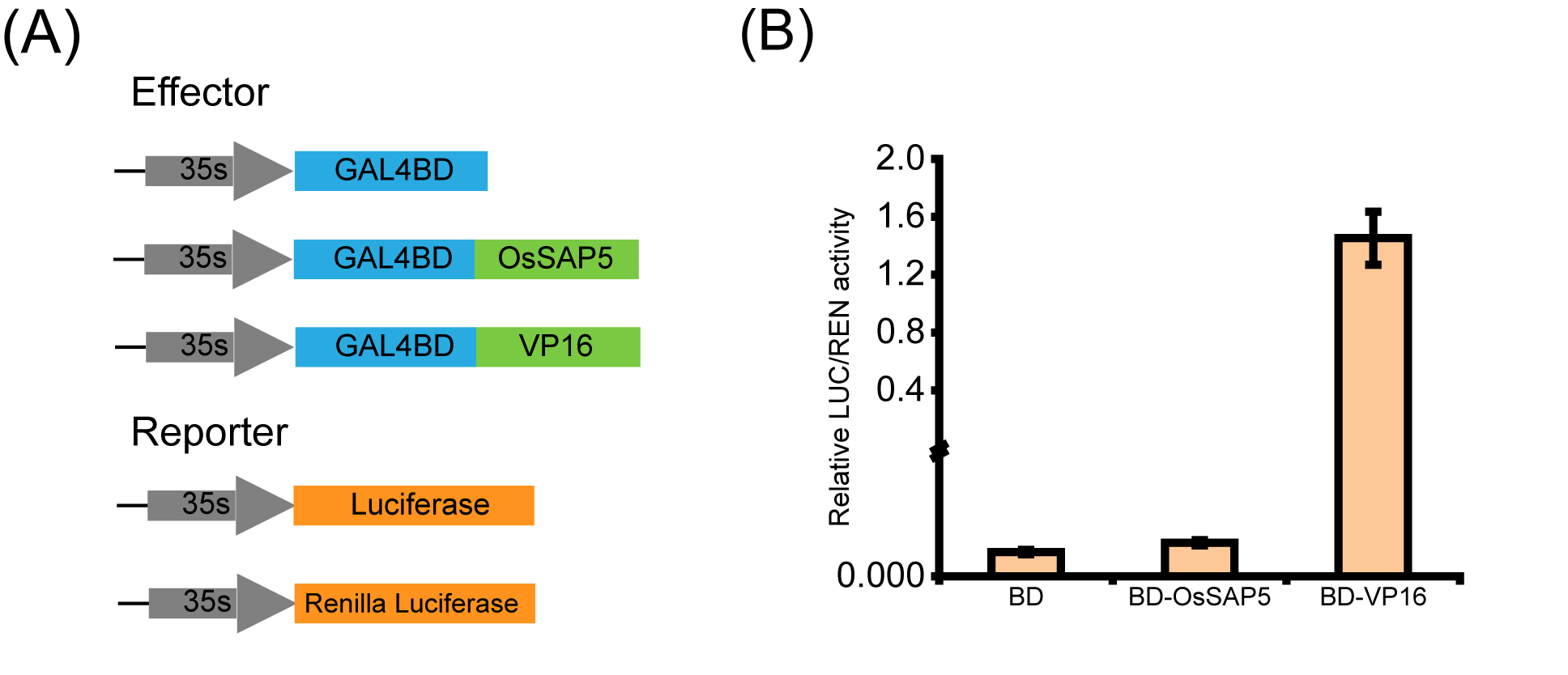

Supplement: Supplementary Figure 1 — Electrophoresis diagram of HYG in OsSAP5 overexpression lines. The positive band is 481 bp; the primer is HYG-F/HYG-R. [file DataSheet1.zip › Supplementary Figure S8.tif]

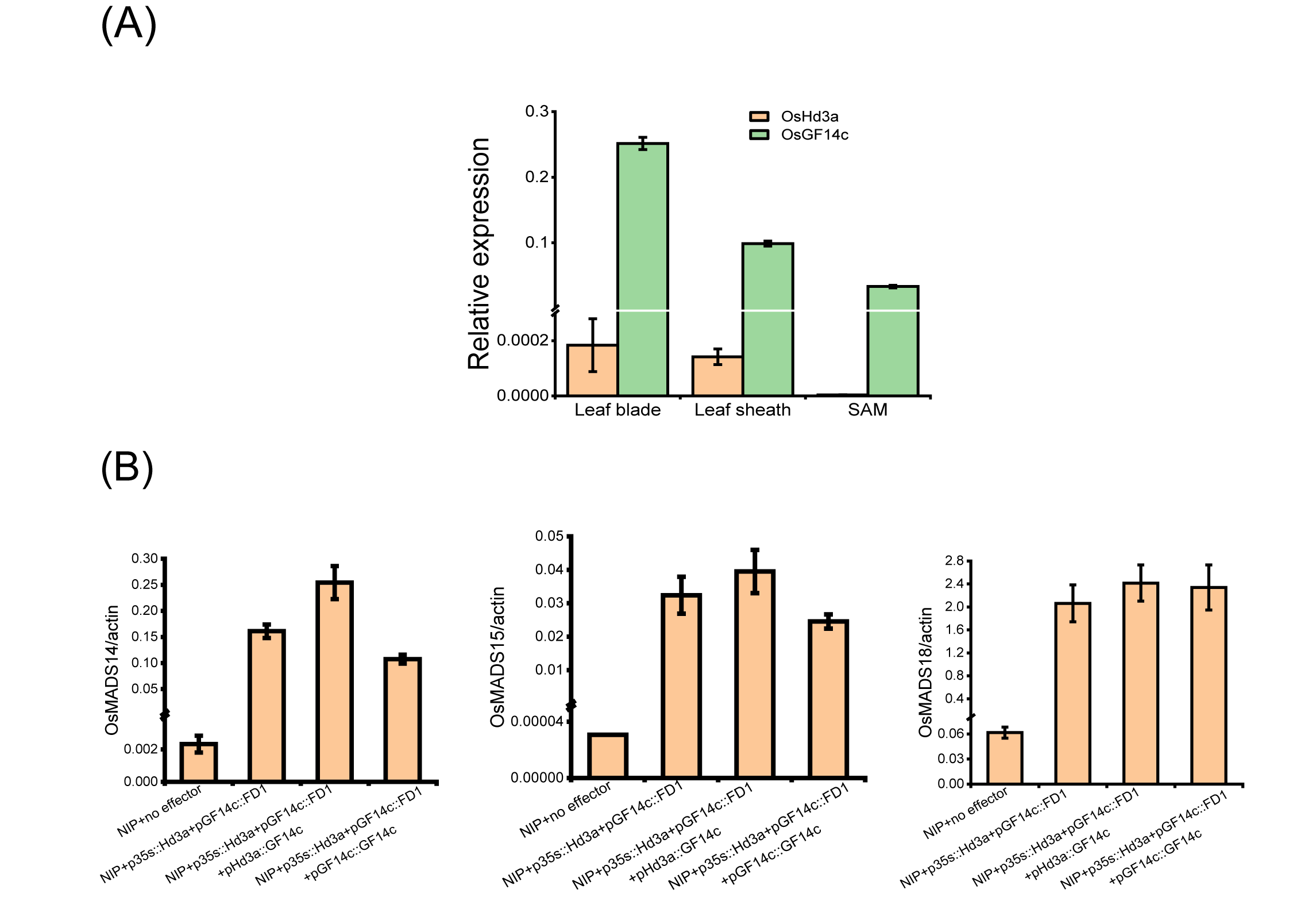

Supplement: Supplementary Figure 1 — Electrophoresis diagram of HYG in OsSAP5 overexpression lines. The positive band is 481 bp; the primer is HYG-F/HYG-R. [file DataSheet1.zip › Supplementary Figure S9.tif]
