# Supplementary material for: Stress-associated protein OsSAP5 regulates rice heading date through interacting with OsGF14c in rice
Source: Front Plant Sci. 2025 Sep 9;16:1589989. doi: 10.3389/fpls.2025.1589989 (PMC12454907; doi:10.3389/fpls.2025.1589989)
Supplement: Supplementary Table 1 — Agronomic traits of NIP, OsSAP5 mutants, and overexpression lines under NSD condition. [file Table1.docx]

**Supplementary Table S1**. Agronomic traits of NIP, *OsSAP5* mutants and overexpression lines under NSD conditions

| Genotype | NSD | | | | | |
| --- | --- | --- | --- | --- | --- | --- |
|  | NIP | *ossap5-1* | *ossap5-2* | NIP | OsSAP5-OE#1 | OsSAP5-OE#3 |
| Plant height（cm） | 62.1±3.59 | 63.7±3.82 | 63.5±3.05 | 66.9±3.57 | 58.1±3.39** | 66.3±2.41 |
| Tiller number | 9.4±2.30 | 8.4±2.60 | 7.6±1.84 | 15.4±5.73 | 20.0±3.73 | 13.6±4.38 |
| Weight/Plot（g） | 14.0±2.79 | 14.9±3.98 | 13.8±3.40 | 21.8±6.27 | 9.2±2.18** | 17.9±3.00* |
| Seed setting percentage（%） | 67.2±13.35 | 77.0±12.30* | 78.4±8.55** | 69.3±8.40 | 27.7±8.63** | 58.1±12.21** |

Note: n=15, **P*<0.05, ***P*<0.01; Student’s *t*-test.
