# Supplementary material for: Stress-associated protein OsSAP5 regulates rice heading date through interacting with OsGF14c in rice
Source: Front Plant Sci. 2025 Sep 9;16:1589989. doi: 10.3389/fpls.2025.1589989 (PMC12454907; doi:10.3389/fpls.2025.1589989)
Supplement: Supplementary file 3 [file Table2.docx]

Supplementary Table S2. Potential interacting partners of OsSAP5 isolated from Co-IP followed by LC-MS/MS analysis.

| Accession | MSU ID | molecular function |
| --- | --- | --- |
| A2YPX2 | LOC_Os07g48020 | responsing to environmental stresses such as wounding, pathogen attack and oxidative stress. |
| A2XFC7 | LOC_Os03g17690 | positively regulating rice spikelet rolerance to chilly on the booting stage |
| A2ZHF9 | LOC_Os12g02370 | Positively regulating tricin-ligin formation |
| A2XL05 | LOC_Os03g49190 | providing recognition signals for specific lipase anchorage in lipolysis during seedling growth |
| A2Y8A0 | LOC_Os06g01390 | playing a role in the synthesis of jasmonic acid in response to wounding |
| B8AL97 | LOC_Os03g57960 | play a role as an initiating endopeptidase in germinating seeds |
| A2YVG3 | LOC_Os08g33370 | OsGF14c-overexpression lines delay heading date |
| Q84JG8 | LOC_Os04g16680 | regulating tiller number in rice |
| B8B4R4 | LOC_Os07g14270 | induced by cold stress |
| B8BHG9 | LOC_Os10g33800 | positively regulating seed development |
| B8AI32 | LOC_Os02g50350 | positively regualting plant tolence to salt stress and drought |
| B8B107 | LOC_Os06g45120 | negatively regulating seed vigour |
| A2XC53 | LOC_Os03g04110 | CEBiP (chitin elicitor binding protein) is essential for chitin signaling in rice. |
| B8B6B6 | LOC_Os07g49400 | positively regualting plant tolence to cold |
| B8BH45 | LOC_Os10g30580 | OsCDC48 maintaining the full ATPase activity |
| A2XAP0 | LOC_Os02g55890 | OVP3 indecing by anoxia |
| B8AGI4 | LOC_Os02g02560 | positively regulating pollen mutatration |
| A2YBK1 | LOC_Os06g15990 | begatively regulating rice resistance to broad-spectrum pathogens |
| A2Y698 | LOC_Os05g42350 | catalytic activity |
| B8AM24 | LOC_Os03g18810 | catalytic activity |
| B8AZS6 | LOC_Os05g43252 | catalytic activity |
| A2YWR6 | LOC_Os08g39300 | catalytic activity |
| A2YP40 | LOC_Os07g44410 | protein binding |
