# Supplementary material for: Stress-associated protein OsSAP5 regulates rice heading date through interacting with OsGF14c in rice
Source: Front Plant Sci. 2025 Sep 9;16:1589989. doi: 10.3389/fpls.2025.1589989 (PMC12454907; doi:10.3389/fpls.2025.1589989)
Supplement: Supplementary file 4 [file Table3.docx]

Supplementary Table S3. Primers used in this study.

| Primers name | Sequences（5‘ to 3’） |
| --- | --- |
| Primers used for CRISPR/Cas9 construction | |
| U-F | CTCCGTTTTACCTGTGGAATCG |
| OsSAP5-U3T1 | TCCTGCCACCTCTGCTCCTCTgccacggatcatctgc |
| OsSAP5-U6aT2 | TGGCGGTCGCGGTAGCACTTCggcagccaagccagca |
| OsSAP5-gRT1 | GAGGAGCAGAGGTGGCAGGAgttttagagctagaaat |
| OsSAP5-gRT2 | AAGTGCTACCGCGACCGCCAgttttagagctagaaat |
| OsGF14c-U3T1 | TTCAGCCTGCTCGGCCAGCgccacggatcatctgc |
| OsGF14c-gRT1 | GCTGGCCGAGCAGGCTGAAgttttagagctagaaat |
| gR-R | CGGAGGAAAATTCCATCCAC |
| Pps-GGL | TTCAGAggtctcTctcgACTAGTATGGAATCGGCAGCAAAGG |
| Pgs-GG2 | AGCGTGggtctcGtcagggTCCATCCACTCCAAGCTC |
| Pps-GG2 | TTCAGAggtctcTctgacacTGGAATCGGCAGCAAAGG |
| Pgs-GGR | AGCGTGggtctcGaccgACGCGTATCCATCCACTCCAAGCTC |
| Primers used for overexpression construction | |
| OsSAP5-MYC-F | ATCCAGATCCAGTGGGATCCATGGCTGAGGAGCAGAGGTG |
| OsSAP5-MYC-R | GCGGCCGCACTAGTAAGCTTGATCTTGTCCTTGAGCTTGT |
| OsGF14c-MYC-F | ATCCAGATCCAGTGGGATCCATGTCTCGGGAGGAGAATGT |
| OsGF14c-MYC-R | GCGGCCGCACTAGTAAGCTTCTGGCCCTCGCAGGCGTCGC |
| Primers used for GFP observation | |
| OsSAP5-  promoter-F | AGTTCCCACTGAATCAAAGGTTGCCTGCTGCCACGCACCA |
| OsSAP5-  promoter-R | CTAGAGGATCCCCGGGTACCGGCTGGCCACCGGCGGCTGT |
| OsSAP5-  promoter-CDS-F | GCCGGTGGCCAGCCGGTACCATGGCTGAGGAGCAGAGGTG |
| OsSAP5-  promoter-CDS-R | CCCTTGCTCACCATGTCGACGATCTTGTCCTTGAGCTTGT |
| Primers used for GUS staining assay | |
| OsSAP5-Gus-F | CCCTTGCTCACCATGGATCCTTGCCTGCTGCCACGCACCA |
| OsSAP5-Gus-R | TAAGGGACTGACCACCCGGGGGCTGGCCACCGGCGGCTGT |
| Primers used for Subcellular localization | |
| OsSAP5-EGFP-F | CGGGGGACGAGCTCGGTACCATGGCTGAGGAGCAGAGGTG |
| OsSAP5-EGFP-R | CCCTTGCTCACCATGTCGACGATCTTGTCCTTGAGCTTGT |
| OsGF14C-EGFP-F | CGGGGGACGAGCTCGGTACCATGTCTCGGGAGGAGAATGT |
| OsGF14C-EGFP-R | CCCTTGCTCACCATGTCGACCTGGCCCTCGCAGGCGTCGC |
| Primers used for qRT-PCR analysis | |
| OsSAP5-qPCR-F | ACGCTCGACCTCTGCTCCAA |
| OsSAP5-qPCR-R | GATGGCGAGGAAGGATGAGT |
| qPCR-MADS14-F | CAACCTCAAACAAGTTCCTC |
| qPCR-MADS14-R | TGCTGCTACATCCTCTATCC |
| qPCR-MADS15-F | TGCTACCCGCCGGTGATGAT |

Supplementary Table S3 Continued

| Primers name | Sequences（5‘ to 3’） |
| --- | --- |
| Primers used for qRT-PCR analysis | |
| qPCR-MADS15-R | CCTCCGATGCGGAGTTGCAC |
| qPCR-MADS18-F | ACCAAGGGCAAGCTCTACG |
| qPCR-MADS18-R | CGCTGGTAGCGTTCAAGG |
| qPCR-MADS34-F | AGCAGCTCCACTGGCTACAAATGA |
| qPCR-MADS34-R | AGGTCGCAGAGTTCATCAAG |
| qPCR-GI-F | ATCGTTCTGCAGGCCGAGA |
| qPCR-GI-R | TCACCAATGCTTCTGGGCTAT |
| qPCR-Ghd7-F | CCAGCGCCACCATCATGTCA |
| qPCR-Ghd7-R | GCCTCCCTCTCCACCATTGC |
| qPCR-Hd1-F | CGTTTCGCCAAGAGATCAG |
| qPCR-Hd1-R | AGATAGAGCTGCAGTGGAGAAC |
| qPCR-Ehd1-F | AACCCGGTCATCCTCCAT |
| qPCR-Ehd1-R | TCATCTCTCACCTCATTTTCT |
| qPCR-GF14b-F | ATGTCGGCACAGGCGGAGC |
| qPCR-GF14b-R | CTCAGAGTCAACCGTCTTGGCC |
| qPCR-GF14c-F | GGTGCAAAGACTGTAGATGTGG |
| qPCR-GF14c-R | TGAGACAATACGCCAGGAGG |
| qPCR-FD1-F | ACAACCTGGAGACCGAGGTG |
| qPCR-FD1-R | AGCACCCTCTGCAGAGTCCT |
| qPCR-Hd3a-F | GCTCACTATCATCATCCAGCATG |
| qPCR-Hd3a-R | CCTTGCTCAGCTATTTAATTGCATAA |
| qPCR-RFT1-F | TGGGTTAGCTGACCTAGATTCAAA |
| qPCR-RFT1-R | GCCAACCACAAGAGGATCGT |
| qPCR-Actin-F | TGGCATCTCTCAGCACATTCC |
| qPCR-Actin-R | TGCACAATGGATGGGTCAGA |
| Primer used for Bimolecular Fluorescence Complementation (BiFC) in tobacco leaves cell | |
| OsSAP5-YCE-F | TGGCGCGCCACTAGTGGATCCATGGCTGAGGAGCAGAGGT |
| OsSAP5-YCE-R | CCCGGGAGCGGTACCCTCGAGGATCTTGTCCTTGAGCTTG |
| OsGF14C-YNE-F | TGGCGCGCCACTAGTGGATCCATGTCTCGGGAGGAGAATG |
| OsGF14C-YNE-R | CCCGGGAGCGGTACCCTCGAGCTGGCCCTCGCAGGCGTCG |
| Primer used for Luciferase Complementation Assay (LCI) in tobacco leaves cell | |
| OsGF14c-nLuc-F | ACGGGGGACGAGCTCGGTACCATGTCTCGGGAGGAGAATG |
| OsGF14c-nLuc-R | CGCGTACGAGATCTGGTCGACCTGGCCCTCGCAGGCGTCG |
| OsSAP5-cLUC-F | TACGCGTCCCGGGGCGGTACCATGGCTGAGGAGCAGAGGT |
| OsSAP5-cLUC-R | ACGAAAGCTCTGCAGGTCGACTCAGATCTTGTCCTTGAGC |
| Primer used for pull-down | |
| OsSAP5-32a-F | CTGATATCGGATCCGAATTCATGGCTGAGGAGCAGAGGTG |
| OsSAP5-32a-R | GCGGCCGCAAGCTTGTCGACGATCTTGTCCTTGAGCTTGT |
| OsGF14c-GST-F | CGCGTGGATCCCCGGAATTCATGTCTCGGGAGGAGAATGT |
| OsGF14c-GST-R | ATGCGGCCGCTCGAGTCGACTTACTGGCCCTCGCAGGCGT |

Supplementary Table S3 Continued

| Primers name | Sequences（5‘ to 3’） |
| --- | --- |
| Primer used for pull-down | |
| OsGF14c-32a-F | CTGATATCGGATCCGAATTCATGTCTCGGGAGGAGAATGT |
| OsGF14c-32a-R | GCGGCCGCAAGCTTGTCGACTTACTGGCCCTCGCAGGCGT |
| OsGF14b-GST-F | CGCGTGGATCCCCGGAATTCATGTCGGCACAGGCGGAGCT |
| OsGF14b-GST-R | ATGCGGCCGCTCGAGTCGACTTACTGCCCCTCGCTGGAGT |
| OsGF14d-GST-F | CGCGTGGATCCCCGGAATTCATGTCGCCGGCGGAGCCGAC |
| OsGF14d-GST-R | ATGCGGCCGCTCGAGTCGACTCACTGATCCCCAGGCTCTT |
| OsGF14e-GST-F | CGCGTGGATCCCCGGAATTCATGTCGCAGCCTGCTGAGCT |
| OsGF14e-GST-R | ATGCGGCCGCTCGAGTCGACTCACTGTCCATCTCCTGATT |
| Primer used for Coimmunoprecipitation in rice protoplasts | |
| OsSAP5-HA-F | ATCCAGATCCAGTGGGATCCATGGCTGAGGAGCAGAGGTG |
| OsSAP5-HA-R | GTAAGCTTGGTACCGAGCTCGATCTTGTCCTTGAGCTTGT |
| OsGF14c-GFP-F | ATCCAGATCCAGTGGGATCCATGTCTCGGGAGGAGAATGT |
| OsGF14c-GFP-R | GTAAGCTTGGTACCGAGCTCCTGGCCCTCGCAGGCGTCGC |
| Primer used for transcriptional activity | |
| gal4-Sap5-F | CGTCTAGAACTAGTGGATCCATGGCTGAGGAGCAGAGGTG |
| gal4-Sap5-R | TTGCGGAgTacccGGGTACCTCAGATCTTGTCCTTGAGCT |
| Primer used for OsMADS expression in rice protoplasts | |
| OsGF14c-promoter-F | AGATTTTGAGACACCTGCAGTCAAACTGTCCATATTTGTC |
| OsGF14c-Promoter-R | TCCCACTGGATCTGGATATCTTTGACCAATTAAGGGATTA |
| Hd3a-promoter-F | AGATTTTGAGACACCTGCAGCGGTTTCAATCAATCACCAG |
| Hd3a-promoter-R | TCCCACTGGATCTGGATATCCGATCTTGCAAAAAACCCTG |
| OsGF14c-promoter-CDS-F | ATCCAGATCCAGTGGGATCCATGTCTCGGGAGGAGAATGT |
| OsGF14c-promoter-CDS-R | GTAAGCTTGGTACCGAGCTCCTGGCCCTCGCAGGCGTCGC |
| OsFD1-promoter-CDS-F | ATCCAGATCCAGTGGGATCCATGGCGATGGAGGACGACGA |
| OsFD1-promoter-CDS-R | GTAAGCTTGGTACCGAGCTCGAATGGCGCGGAGAGCACCC |
| Hd3a-CDS-F | ATCCAGATCCAGTGGGATCCATGGCCGGAAGTGGCAGGGA |
| Hd3a-CDS-R | GTAAGCTTGGTACCGAGCTCGGGGTAGACCCTCCTGCCGC |
